# Quinoidal Azaacenes: 99 % Diradical Character

**DOI:** 10.1002/anie.201915977

**Published:** 2020-04-28

**Authors:** Sebastian N. Intorp, Manuel Hodecker, Matthias Müller, Olena Tverskoy, Marco Rosenkranz, Evgenia Dmitrieva, Alexey A. Popov, Frank Rominger, Jan Freudenberg, Andreas Dreuw, Uwe H. F. Bunz

**Affiliations:** ^1^ Organisch-Chemisches Institut Ruprecht-Karls-Universität Im Neuenheimer Feld 270 69120 Heidelberg Germany; ^2^ Interdisziplinäres Zentrum für Wissenschaftliches Rechnen Ruprecht Karls-Universität Heidelberg Im Neuenheimer Feld 205 69120 Heidelberg Germany; ^3^ Center of Spectroelectrochemistry Leibniz Institute for Solid State and Materials Research (IFW) Dresden Helmholtzstraße 20 01069 Dresden Germany; ^4^ Centre for Advanced Materials Ruprecht-Karls-Universität Im Neuenheimer Feld 225 69120 Heidelberg Germany

**Keywords:** acenes, heteroaromatics, radicals, structure elucidation, synthetic methods

## Abstract

Quinoidal azaacenes with almost pure diradical character (*y=*0.95 to *y=*0.99) were synthesized. All compounds exhibit paramagnetic behavior investigated by EPR and NMR spectroscopy, and SQUID measurements, revealing thermally populated triplet states with an extremely low‐energy gap Δ*E*_ST′_ of 0.58 to 1.0 kcal mol^−1^. The species are persistent in solution (half‐life≈14–21 h) and in the solid state they are stable for weeks.

## Graphical abstract

**Best of two worlds**: Lateral fusion of azaacenes onto a quinoidal system furnishes stable diradicals with a radical character (*y*) of up to 0.99. All compounds exhibit paramagnetic behavior as determined by EPR and NMR spectroscopy and SQUID measurements, revealing thermally populated triplet states with an extremely low‐energy gap Δ*E*_ST_ of 0.58 to 1.0 kcal mol^−1^. The species are persistent in solution (half‐life≈14–21 h), and they are stable for weeks in the solid state.

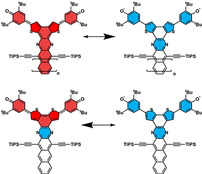

Organic open‐shell molecules, that is, diradicals and diradicaloids, have unique magnetic and electronic properties. Early examples were Tschitschibabin's^1^ and Thiele's hydrocarbons,^2^ but the topic has garnered significant additional impact recently since the groups of Haley,^3^ Wu,^4^ Chi,^5^ Casado,^6^ and Navarette,^7^ among others,^8^ have re‐developed and expanded this field. While the authors deploy different structures such as π‐extended indenes, fluorenes, rylenes, or zethrenes, the essence of most of the approaches is the formation of diradicals as a result of aromatization of a formal quinoidal subsystem, creating additional Clar sextets as a driving force. Energetically, this process is often more favorable than electron‐pairing and as a consequence, either diradicals or diradicaloids form.^9^, ^10^, ^11^ As a measure of diradical character, the contributions of quinoidal closed‐shell and aromatic open‐shell structures is quantum chemically evaluated in terms of the diradical character *y*, where *y=*0 represents a purely closed‐shell molecule and *y=*1 represents a pure diradical. Whether these diradicals display a triplet or singlet ground state is a difficult question and depends on overlap integral of orbitals and their spatial separation.^12^, ^13^

Diradicals and diradicaloids are of great current interest because of their unique structural and conceptional value,^14^ but also because of their potential applications in thermoelectric generators^15^ and spintronics.^16^ In the literature, diradicals having triplet ground states^17^ and singlet ground states with thermally accessible low‐lying triplet states are described. The highest reported diradical characters are up to 0.99,^18^ however, only few Kekuléan species with high diradical character are stable and persist under laboratory conditions.

Two main strategies alter the extent of diradical character: 1) extension of quinoidal cores between the radical centers, as demonstrated by elongation of a quinodimethane‐based rylene system, increasing the diradical character almost to unity in the formal hexamer;^18^ or 2) exploiting regioisomerism as shown for the indenofluorenes3b (Figure 1).

**Figure 1:**
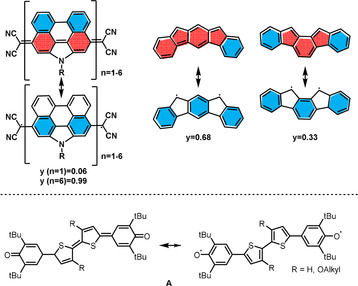
Strategies to enhance the diradical character3b, 18 and structure of the quinoidal oligothiophene **A** (only one geometric isomer depicted).^19^

Sterically encumbered phenoxy‐groups^20^ form stable radicals in non*‐*Kekulé structures^21^ and introduce diradical character to Kekuléan quinoidal systems,^22^ the simplest of which are the oxo‐analogues of Thiele's and Tschitschibabin's hydrocarbons.^23^

The degree of diradical character, however, largely depends on the π‐system bridging the two phenoxy species. Calculations from our group showed the quinoidal thiophene **A** (R=H) to exhibit a diradical character of 18 %, consistent with the 14 % reported in literature^19^ (Figure 1). Its quinoidal ground state was further stabilized by alkoxy substitution. How can a substantial increase in the radical character of such a system be achieved? Herein, we merge azaacenes^24^ with two bis(thiophene)‐based quinoidal systems and investigate the influence of regioisomerism and acene length on the diradical character *y*. In contrast to the literature, an increase of *y* is not achieved by varying the (*para*‐)quinoidal system in between the two radical centers, but by lateral annulation of *ortho*‐quinoids, which results in highly proaromatic azaacene‐quinones (**1 a**–**c,** Scheme 1). We demonstrate that both the lateral attachment of azaacenes and the regiochemistry of the bithiophene unit determine the percentage of diradical character (Scheme 1), qualitatively described by the difference in numbers of Clar sextets of the resonance structures. Our concept allows an increase from *y*=0.26 for the azaacene **2 c** to almost quantitative diradical character for the largest representatives of the regioisomeric series **1**.

**Scheme 1:**
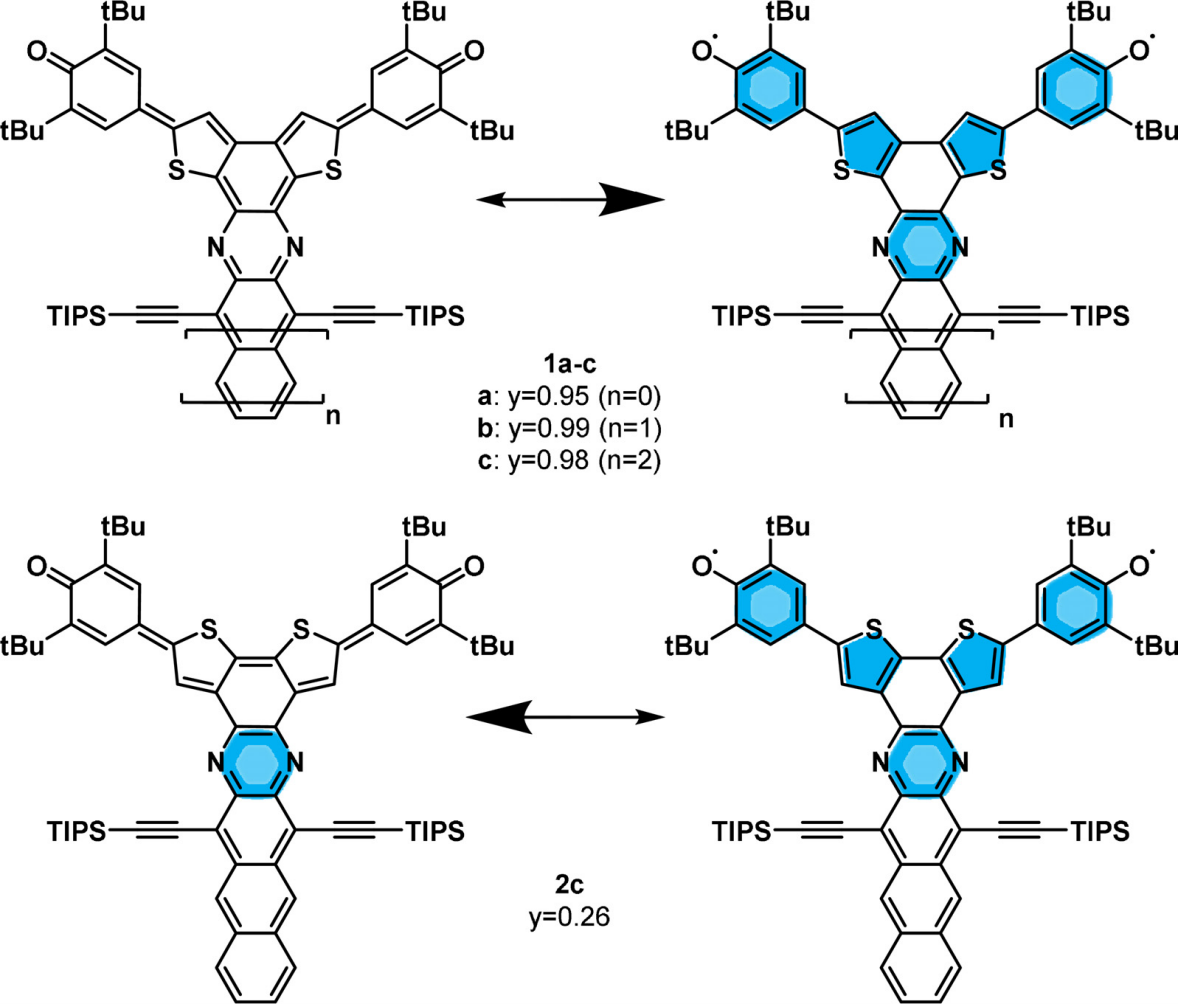
Azaacenequinones (**1 a**–**c**) and its regioisomer **2 c**. Resonance structures of the closed‐shell quinoid and open‐shell aromatic and diradical systems.

The syntheses of **1** and **2 c** are similar, and we will only discuss the preparation of **1 a**–**c**, and refer to the Supporting Information for **2 c** starting from the isomeric dione **7**. The diamines **3 a**–**c** are condensed with the dibromodiketone **4** (Scheme 2).^25^ Coupling of **5 a**–**c** with 3,5‐di‐*tert*‐butyl‐4‐(trimethylsilyloxy)boronic acid under standard Suzuki conditions gives the corresponding **6 a**–**c** as colored crystalline materials. Potassium cyanoferrate(III) or lead(IV) oxide furnish the quinone/diradical form **1 a**–**c**. All of the quinones, including **2 c**, are stable in the solid state for at least eight weeks when stored at 4 °C (N_2_). Under ambient conditions, the half‐IR‐life, t_1/2_, in dilute toluene solution amounts to 21 hours (**1 a**), 14 hours (**1 b**), 19 hours (**1 c**), and 104 hours (**2 c**), corroborating their relative diradical characters. The absorption spectra (Figure 2) show a bathochromic shift for increased π‐system lengths with a rather long absorption onset from **1 a** to **1 c** and the effect of the different conjugation pathways^26^ for regioisomers **1 c** and **2 c** and the phenolic precursors **6 c** and **9 c** (see the Supporting Information). Proton nuclear magnetic resonance hints at the (partial) diradical character of **1 a**–**c** and **2 c**. Whereas **1 a**–**c** do not show any resonances above −90 °C, highlighting their globally delocalized diradical character, proton resonances of **2 c** at ambient temperature are observed, but only for those belonging to the azaacene moiety far away from the radical centers and residing spin density (see Figure S9 in the Supporting Information). Upon cooling, signals attributable to the thiophene and two distinguishable phenyl groups emerge. X‐ray single‐crystal diffraction however unambiguously identify **1 c**/**2 c**, grown by slow diffusion of a toluene solution into acetonitrile (Figure 3 and the Supporting Information).^27^ A pronounced quinoidal character is observable through single/double‐bond length alternations for **2 c** (see Figure S7), alterations are less pronounced for **1 c**. Especially its C−O bond length with 1.28 Å is longer than for **2 c** (1.21 Å), as it would be expected for a weaker C−O single bond. In both cases, the packing motifs are dominated by the π–π interaction of the azaacene and do not show interaction of radical centers (see Figure S6b). The IR spectra (see Figure S4) are also instructive. **2 c** displays an intense C=O band at 1573 cm^−1^, while **1 c** displays only weak bands between 1500 and 1800 cm^−1^, suggesting the absence of carbonyl groups.

**Figure 2:**
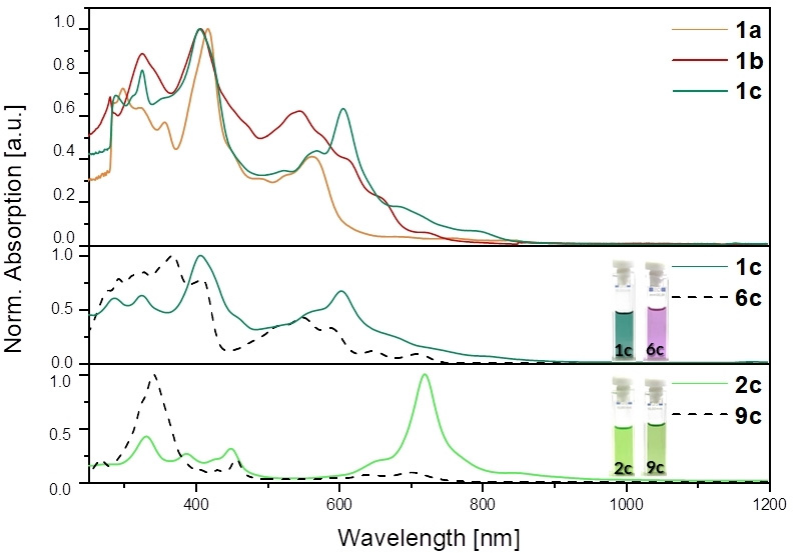
Top: Absorption spectra of **1 a**–**c** in toluene. Middle and Bottom: Absorption spectra of the regioisomers **1 c** and **2 c**, their phenolic precursors **6 c** and **9 c**, and photographs of solutions.

**Figure 3:**
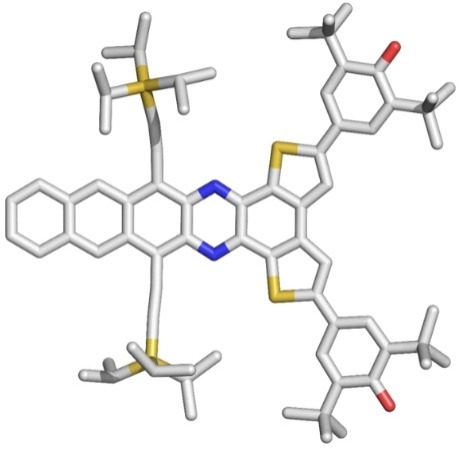
X‐ray crystal structure of **1 c**. Hydrogen atoms were omitted for clarity.

**Scheme 2:**
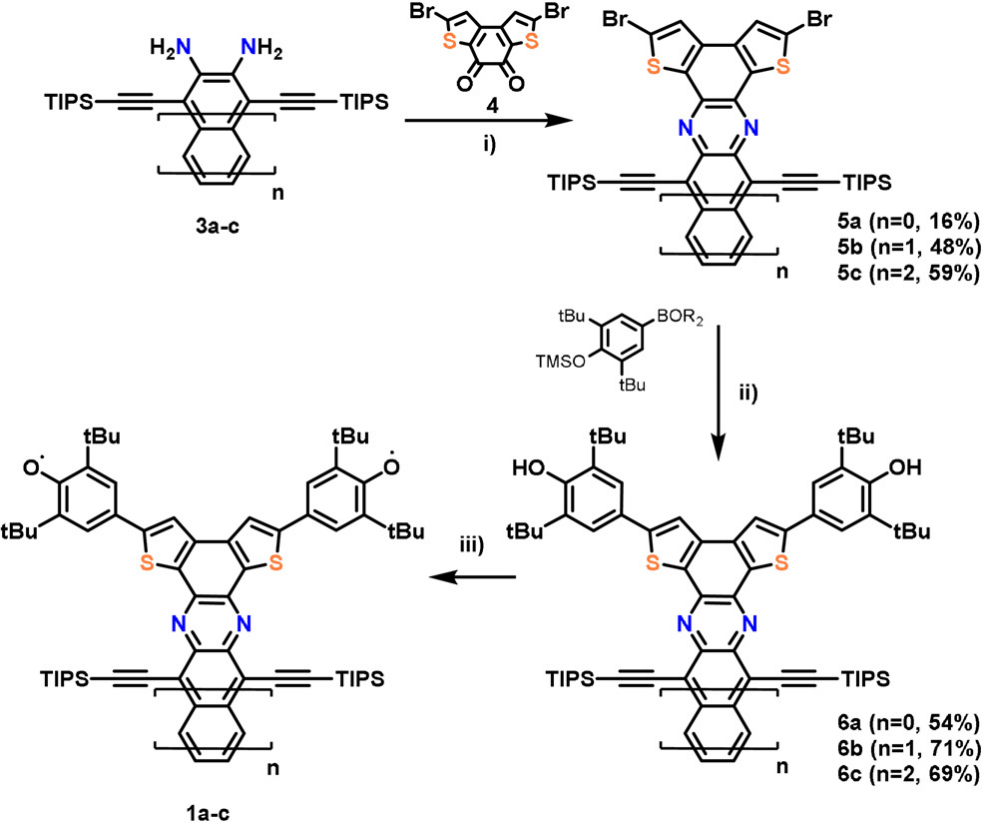
Synthesis of **1 a**–**c**. i) AcOH, 80 °C, reflux; ii) Pd(PPh_3_)_4_, Na_2_CO_3_, THF/H_2_O (10:1, v/v), 60 °C; iii) [K_3_Fe(CN)_6_], KOH, THF/H_2_O (1:1, v/v), RT.

Cyclic voltammetry (Figure 4) reveals a reduction at low potentials for **1 a**–**c** (*E* ^red^ of ca. −0.63 V vs. Fc^+^/Fc), attributed to the consecutive generation of radical anion and dianion of the formerly quinodal system.^28^ For **2 c** the same reduction processes are observed, however, primary reductions are located at more negative potentials (−0.76 V). All systems show an additional reversible reduction of the azaacene moiety between −1.31 and −1.44 V.

**Figure 4:**
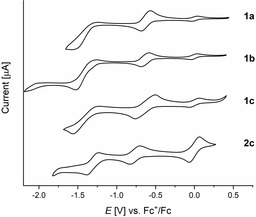
Cyclic voltammetry of **1 a**–**c** and **2 c** using a glassy carbon working electrode, a platinum/titanium wire auxiliary electrode, a silver wire reference electrode, a 0.1 m NBu_4_PF_6_ solution in degassed dry dichloromethane.

The diradical character was calculated by a population analysis of natural orbitals at the DFT/CAM‐B3LYP/6–311G(d,p) level of theory (see also the Supporting Information for a comparison of different functionals). The regioisomers **1** displays high diradical character (**1 a**: *y*_0_=0.96, **1 b**: *y*_0_=0.99 and **1 c**: *y*_0_=0.98). Comparing **1 a**–**c**, with almost pure diradical character, to its regioisomer **2 c** (*y*_0_=0.26), suggests that regioisomerism significantly impacts radical character, qualitatively explained by the different number of Clar sextets formed (four for **2 c**, five for the series of **1**) when going from the proaromatic to the diradical resonance structure (see Figure 1).

Spin‐flip (SF‐DFT) calculations^29^ (B3LYP/6–311G(d,p) level) and comparison of the *M_S_*=0 states suggest open‐shell singlet ground states for **1 a**–**c** with small energy gaps between 0.02 kcal mol^−1^ (**1 a**) and 0.76 kcal mol^−1^ (**1 c**) to the triplet state. **2 c** shows a closed‐shell singlet ground state with an energy gap of 15.2 kcal mol^−1^. Figure 5 displays spin‐density distribution of the triplet state of **1 a**–**c**. It is distributed over the quinonidal system and the acene. In **2 c** the spin density is solely distributed over the quinoidal system. The tetracenylene is a separate aromatic entity, already equipped with a Clar sextet. NICS(1)zz calculations (B3LYP/6–311++ G(d,p) level of theory for the triplet state of **1 a**–**c** and the closed‐shell singlet for **2 c**, Figure 5) indicate negative, aromatic chemical shifts for the acene backbone and the thiophene rings as it would be anticipated for the diradical form for **1 a**–**c**. As expected, the formal triphenylene‐like central ring connecting the thiophenes depicts less pronounced aromaticity or even slight antiaromaticity in case of **2 c**.^30^ The quinoidal nature of the closed‐shell form of **2 c** is reflected by its smaller, non‐aromatic NICS values. In both cases, the phenoxy groups are non‐aromatic—a finding in accordance with literature.^31^ HOMA values (see Figure S15) confirm the results of the NICS calculations.

**Figure 5:**
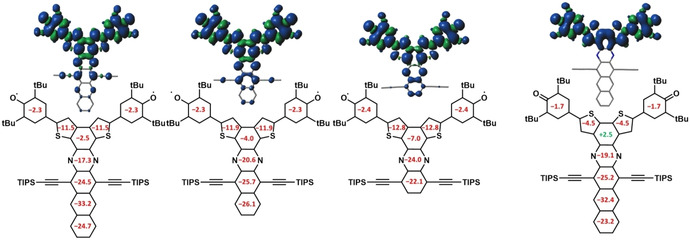
Calculation of spin distribution for the triplet state and NICS(1)zz values calculated at the B3LYP/6–311++G(d,p) level of theory values for the triplet state for **1 a**–**c**. For **2 c** the spin distribution is shown for the triplet state, the NICS(1) values for for the closed‐shell singlet ground state.

The fine‐structured EPR spectra of **1 a**–**c** and **2 c** (Figure 6; see Figure S10c) show signals with *g*‐values of 2.0043. For **1 c** in solid state (Figure S10a), the paramagnetic signal is saturated at room temperature [variable‐temperature EPR (VT‐EPR), Figure 6, top inset; see Figure S10c], that is, a thermally populated triplet state is observed. For **2 c** we observe an exponentially increasing paramagnetic signal beyond 350 K and a faster decreasing of signal intensity in comparison to **1 c** upon cooling (see Figure S10b). Fitting of the I_EPR_*T–*T* data with the Bleaney–Bowers equation furnished the singlet triplet Δ*E*_ST_ gaps of 0.42 kcal mol^−1^ (**1 a**), 0.28 kcal mol^−1^ (**1 b**), 0.30 kcal mol^−1^ (**1 c**), and 2.32 kcal mol^−1^ (**2 c**), in good agreement with the theoretical data, reflecting the trend of the extent of the diradical character. Data of SQUID‐measurements is presented in Figure S11a and supports the previous findings. The *χT*–*T* plot shows an increasing signal for higher temperatures as it would be expected for a singlet ground state. Comparison with simulations of the Bleaney–Bowers equation shows a reasonable agreement with a Δ*E*_ST_ of in the range of 0.1–0.5 kcal mol^−1^, thus confirming the results from VT‐EPR.

**Figure 6:**
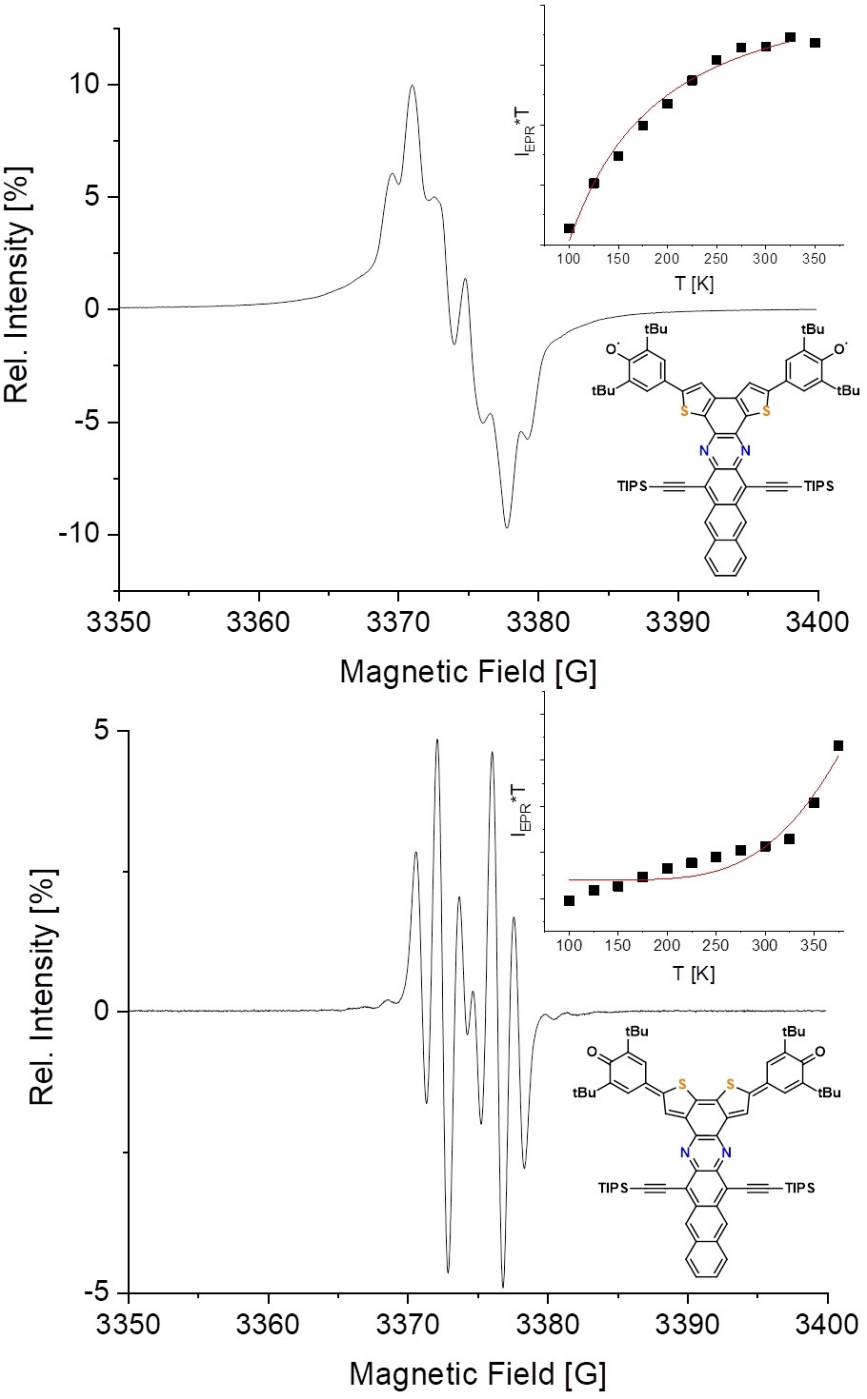
EPR spectra of **1 c** (top) and **2 c** (bottom) in toluene at room temperature with VT‐EPR intensities of solid‐state samples (inset).

How can this large increase of diradical character for the series **1** be rationalized in comparison with quinoidal thiophenes **A** and **2 c**? All compounds include two conjugated phenoxy groups, which aid in creating stable radicals. The reason for the increase in radical character is somewhere else. Figure 7 depicts four model compounds, **1M**/1M* and **2M**/2M*, for the regioisomers. Benzannulation of **A** creates **2M** with increasing radical character (*y*_0_=0.18 vs. *y*_0_ *=*0.39) because of the formed *para*‐quinodimethane. However, further pyrazannulation to 2M* reduces *y* (*y*_0_=0.32) and increases the singlet–triplet gap, as would be expected given the delocalization of the double bond into the Clar sextet of the annulated ring. This behavior is also reflected in the spin‐density distribution of 2M* being more pronounced on the junction of the two thiophenes (see Figure S13). Increasing the appended acene in size and thus delocalization decreases *y* and increases Δ*E*_ST_ (see Figure S14).

**Figure 7:**
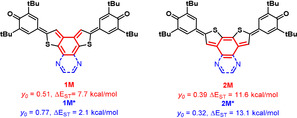
The model compounds **1M**, 1M* and **2M**, 2M* with *ortho*‐quinodimethane and *para*‐quinodimethane substructures.

For the regioisomers, benzannulated **1M** represents an *ortho*‐quinodimethane with a more pronounced disjunction of beta‐connected bithiophenylenes.^32^ Thus, its diradical character is higher than that of the *p*‐QDM (*y*_0_=0.51).

Upon further pyrazannulation, radical character increases (*y*_0_=0.78), and the singlet–triplet gap decreases because of the decreasing stability of the extended annulated quinoidal system (also reflected in the decreasing stabilities in the series furan, isobenzofuran, and isonaphtofuran,^33^ the latter being too instable for isolation), providing a driving force to the diradical form and rearomatization of both the annulated system as well as the thiophenylene‐based system. Radical character exhibits a saturation and a maximum for **1 b** with *y*_0_=0.99 (the one of **1 c** still is calculated to 0.98) and decreases upon annulation of even larger acenes. We attribute this trend to acenes themselves exhibiting diradical character with increasing size,^34^ as a result of the formation of two Clar sextets instead of one as the driving force. Indeed, this behavior is reflected in the increasing tetraradical character of the larger species (see the Supporting Information for resonance structures). Calculated energy gaps confirm this trend.

We disclose a novel concept for the modulation of the diradical character (up to 99 %) and concomitantly small singlet–triplet gaps. The modulation is achieved by the position of the sulfur in the benzodithiophene unit and the length of the attached azaacene. The regioisomers display different conjugation pathways, which lead to either a fully integrated (**1**) or an electronically disjunct (**2**) system. We also find an influence of the diazaacene length on the diradical character of **1**, with a theoretical maximum for the tetracene derivative **1 b**. VT‐EPR measurements confirm this finding with the smallest singlet–triplet energy gap. The entire series **1 a**–**c** represents persistent diradicals at ambient temperature.

## Conflict of interest

The authors declare no conflict of interest.

## Supporting information

As a service to our authors and readers, this journal provides supporting information supplied by the authors. Such materials are peer reviewed and may be re‐organized for online delivery, but are not copy‐edited or typeset. Technical support issues arising from supporting information (other than missing files) should be addressed to the authors.

Supplementary
