# Supplementary material for: Quinoidal Azaacenes: 99 % Diradical Character
Source: Angew Chem Int Ed Engl. 2020 Apr 28;59(30):12396–401. doi: 10.1002/anie.201915977 (PMC7384067; doi:10.1002/anie.201915977)
Supplement: Supplementary file 1 — Supplementary [file ANIE-59-12396-s001.pdf]

Angewandte Chemie
Eine Zeitschrift der Gesellschaft Deutscher Chemiker

Supporting Information

Quinoidal Azaacenes: 99 % Diradical Character

Sebastian N. Intorp, Manuel Hodecker, Matthias Müller, Olena Tverskoy, Marco Rosenkranz, Evgenia Dmitrieva, Alexey A. Popov, Frank Rominger, Jan Freudenberg, Andreas Dreuw, and Uwe H. F. Bunz**

anie_201915977_sm_miscellaneous_information.pdf

1. Synthesis of Regioisomer **2c** S2
2. UV-Vis Absorption S2
3. Photographs S3
4. IR Spectroscopy S4
5. Cyclic Voltammetry S5
6. X-ray Single Crystal Analysis S6
7. Bond Length Alternation S7
8. ^{1}H NMR at -90°C S7
9. VT-NMR of **2c** S8
10. EPR Spectroscopy S9
11. SQUID Magnetometry S13
12. Stability in Toluene S14
13. Calculations of Diradical Character S15
14. Evaluation of Structur Property Relationships S16
15. HOMA Calculations S18
16. NICS-XY-Scans S19
17. General Remarks S21
18. Synthetic Procedures S22
19. ^{1}H NMR and ^{13}C NMR Spectra S25
20. Mass Spectra S29
21. Crystallographic Data S33

1. Synthesis of Regioisomer 2c

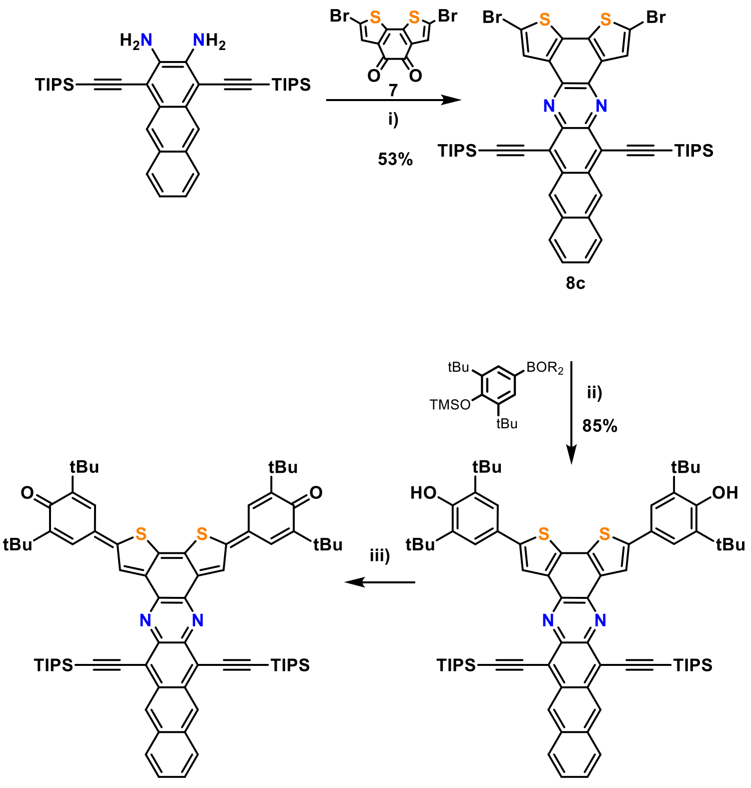

Figure S1 Synthesis of **2c**. i) AcOH, 2 d, reflux; ii) $Pd(PPh_3)_4$, Na_2CO_3, THF/H_2O (10:1, v/v), 60 °C; iii) $[K_3Fe(CN)_6]$, KOH, THF/H2O (1:1, v/v), rt.

2. UV-Vis Absorption

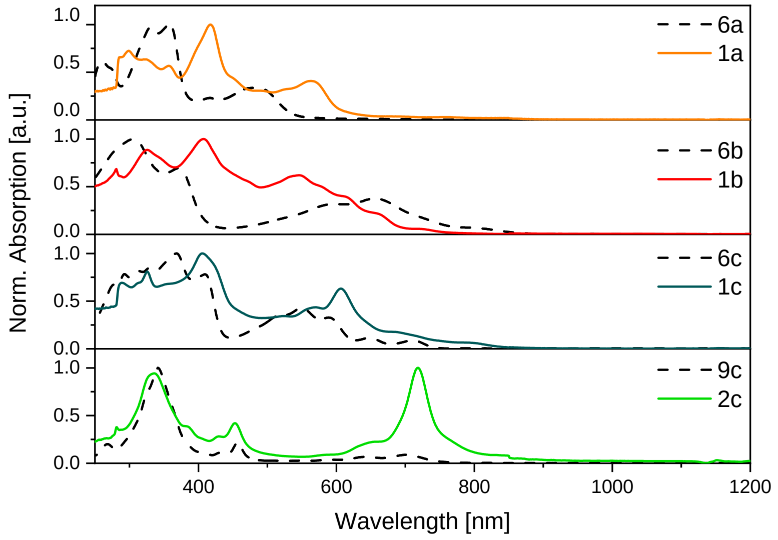

Figure S2: UV-Vis absorption spectra of **AAQs** in chloroform at room temperature.

3. Photographs

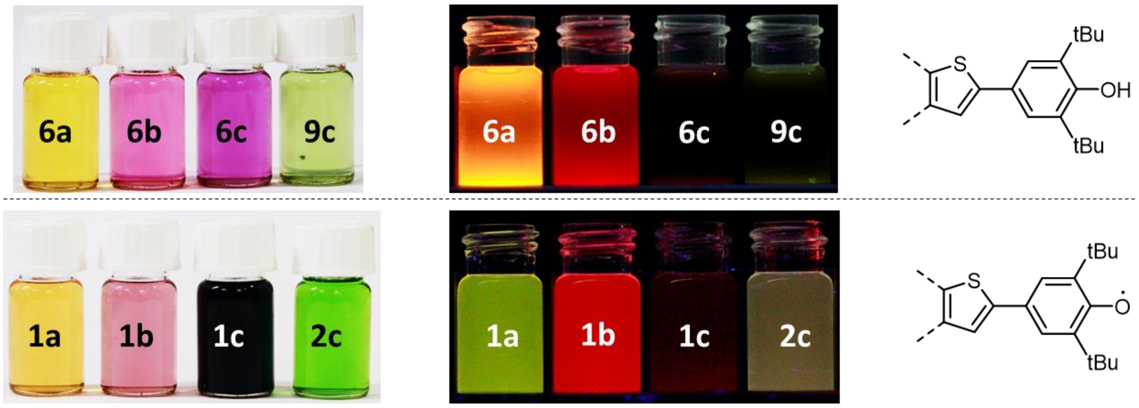

Figure S3: Photographs of phenols **6a-c**, **9c** and diradicals **1a-c**, **2c** in dichloromethane under daylight (left) and under irradiation with a 254 nm lamp (middle).

Table S1. Optical properties of phenols and diradicals in chloroform at room termperature.

	Abs_{max}^{1}[nm]	Abs_{max}^{2}[nm]	Abs_{max}^{3}[nm]	Onset[nm]
**1a**	418	562	-	787
**1b**	409	547	-	827
**1c**	405	607	-	848
**2c**	337	719	-	832
**6a**	358	550	-	712
**6b**	352	526	-	734
**6c**	368	481	709	754
**9c**	343	456	703	783

4. IR Spectroscopy

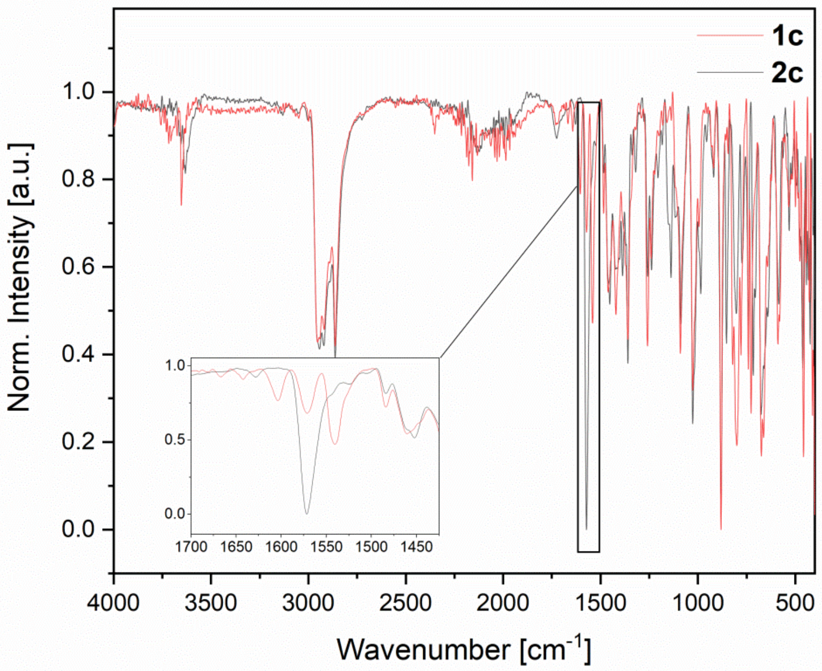

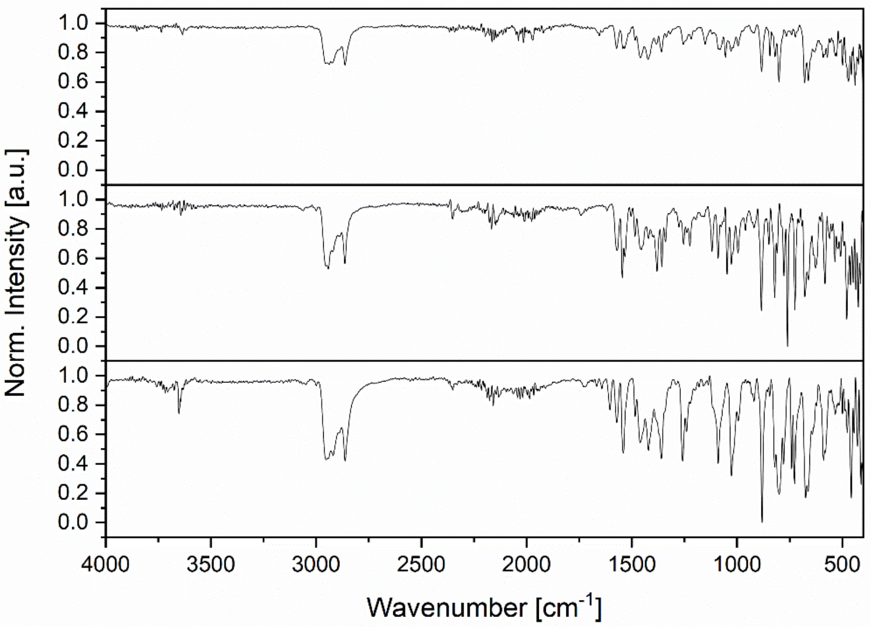

Figure S4. IR spectroscopy of a) **1a-c** and comparison between **1c** and **2c** with inset at 1400-1700 cm^{-1}.

5. Cyclic Voltammetry

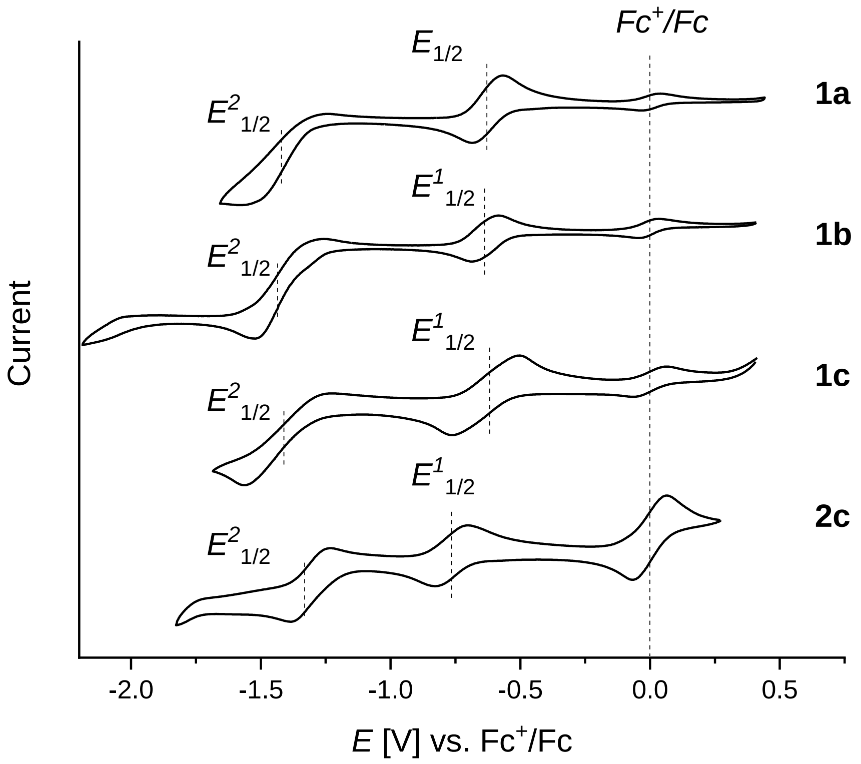

Figure S5. Cyclic voltammogram of **1a-c** measured using a glassy carbon working electrode in DCM containing NBu_4PF_6 (0.1 M) as supporting electrolyte at 50 mV/s. The redox event at 0 V corresponds to ferrocenium/ferrocene (Fc+/Fc) couple.

Table S2. Reduction potentials for **1a-c** and **2c.**

	$E^1_{1/2}$ [V]	$E^2_{1/2}$ [V]
**1a**	−0.63	−1.42
**1b**	−0.63	−1.44
**1c**	−0.63	−1.41
**2c**	−0.76	−1.31

6. X-ray Single Crystal Analysis

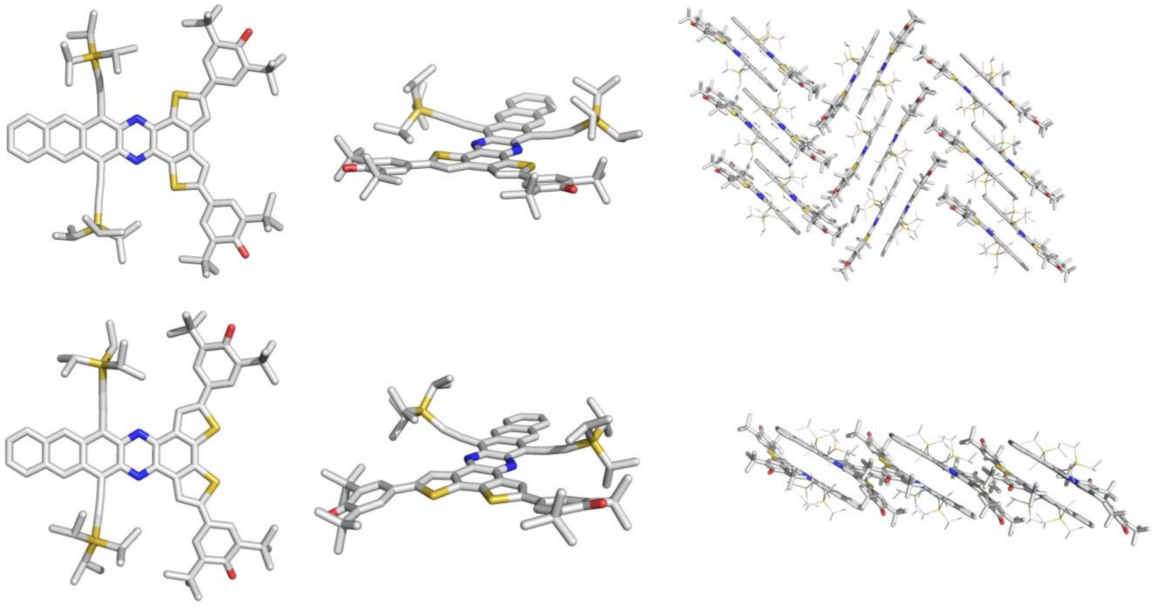

Figure S6a. Crystal structures of **1c** (top) and **2c** (bottom).

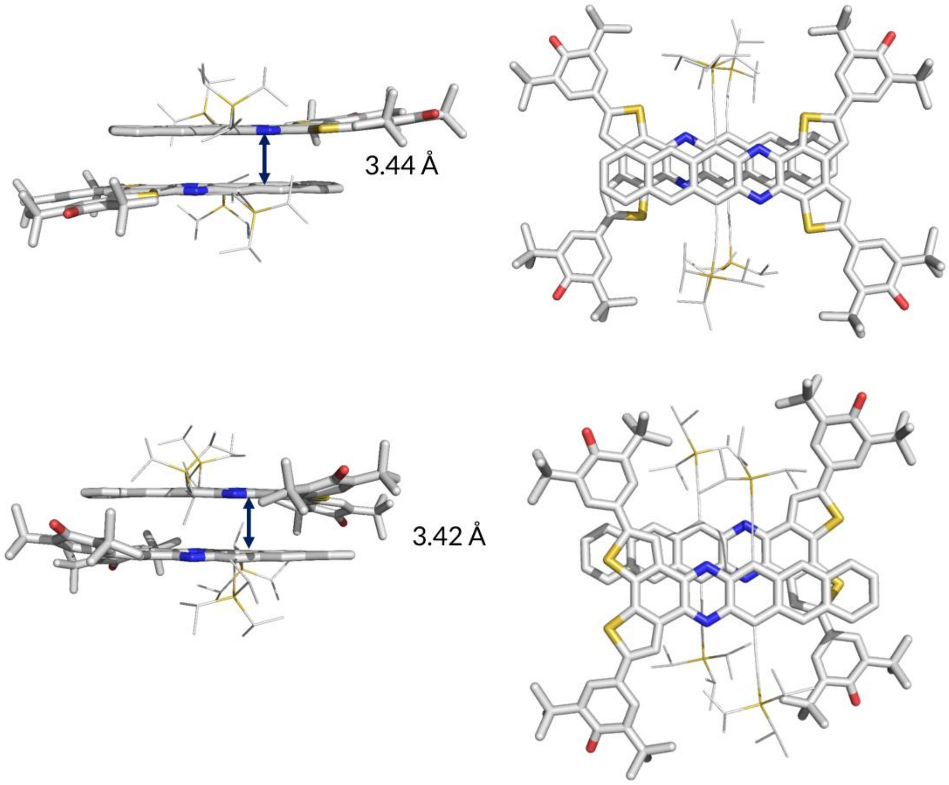

Figure S6b. Dimer formation of **1c** and **2c** in the single crystal. Distances were measured between averaged planes of acene backbones.

7. Bond Length Alternation

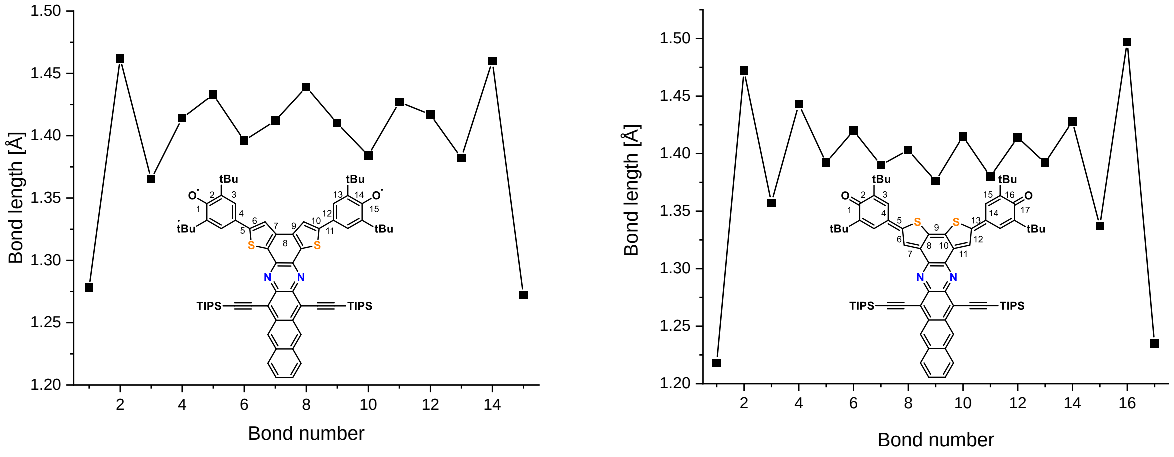

Figure S7 Bond length alterantion along the quinoid structure in **1c** and **2c**.

8. ^{1}H NMR of Isomers 1a-c at -90°C

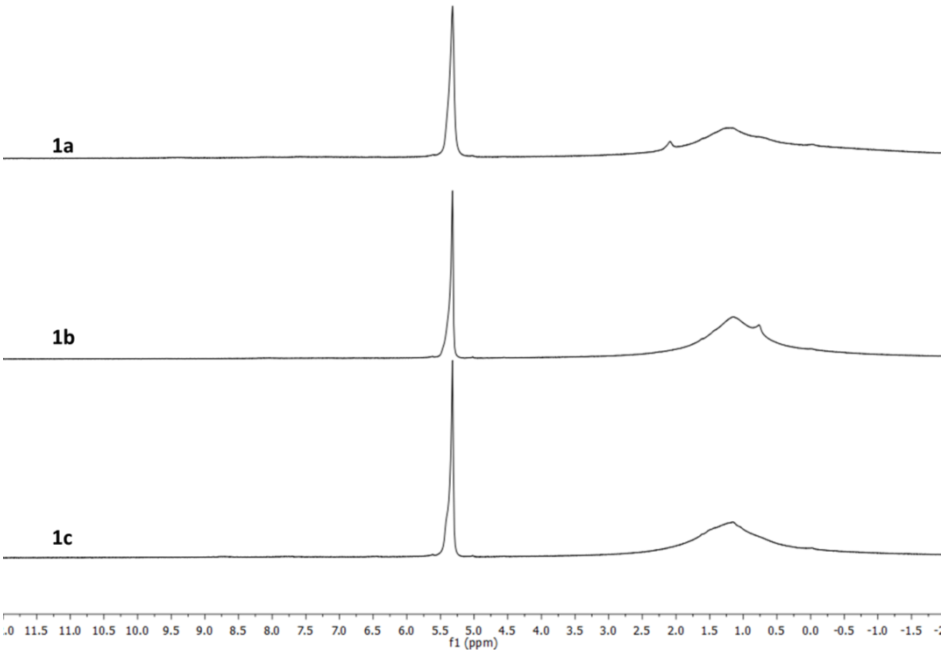

Figure S8. ^{1}H NMR spectra of **1a-c** in CD_2Cl_2 at -90°C.

9. VT-NMR of Isomer 2c

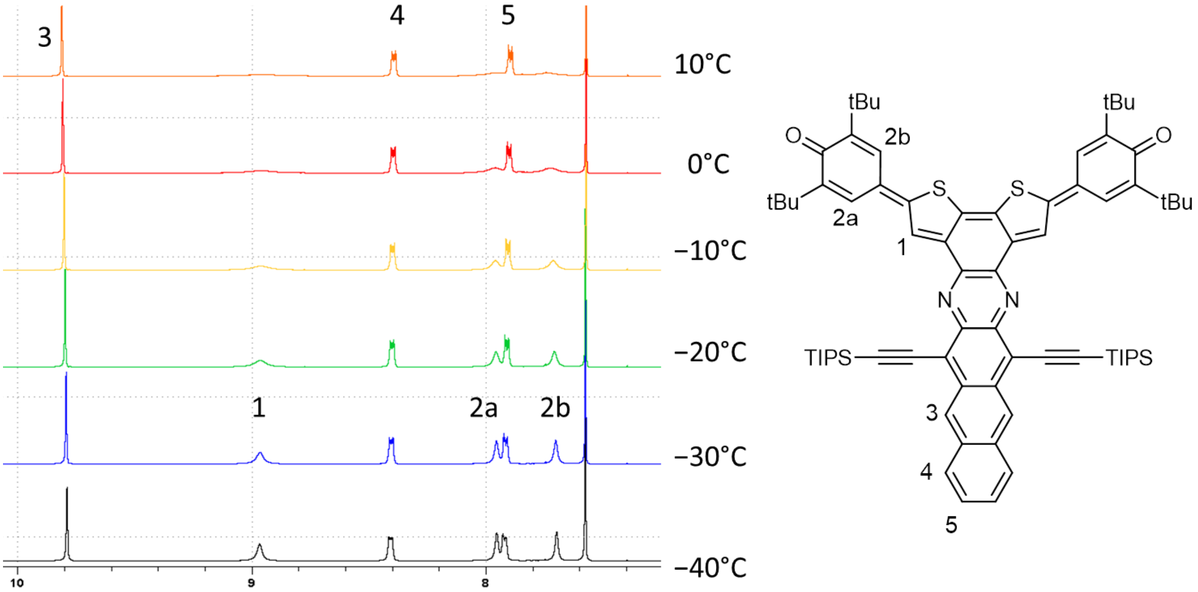

Figure S9. ^{1}H VT-NMR **2c** with emerging signals for protons at the quinoid system (**1**, **2a,b**) at lower temperatures.

10. EPR Spectroscopy

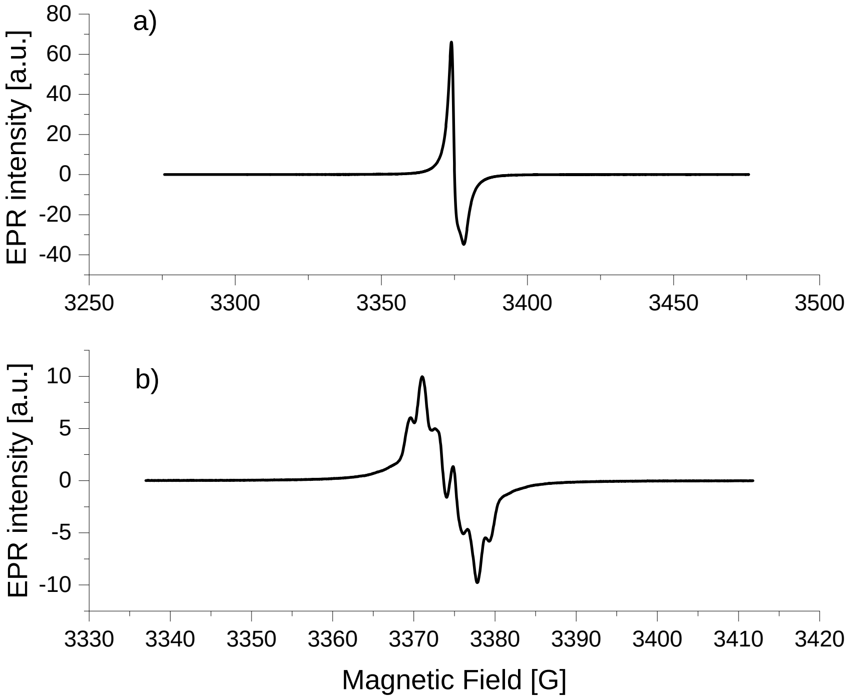

Figure S10a: Solid state EPR (top) and in toluene (bottom) of **1c** at room temperature.

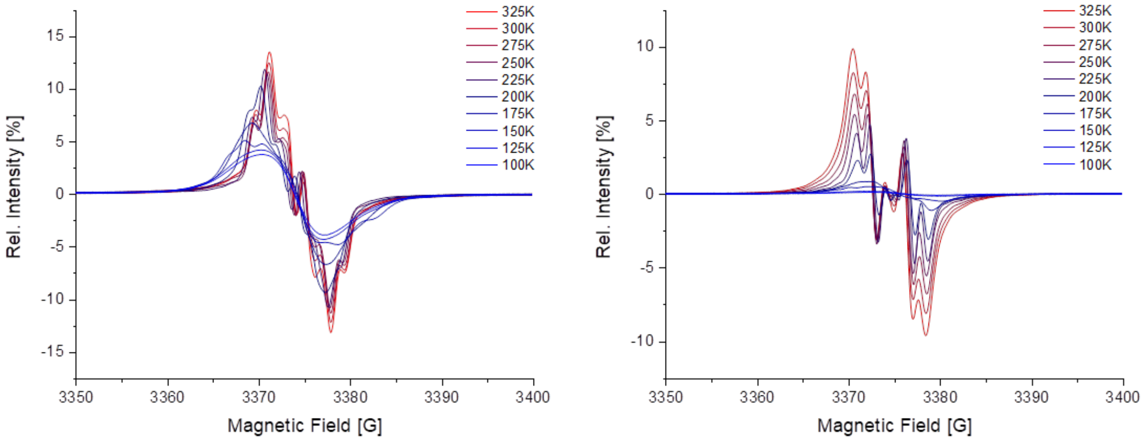

Figure S10b: EPR spectra of **1c** and **2c** in toluene at different temperatures.

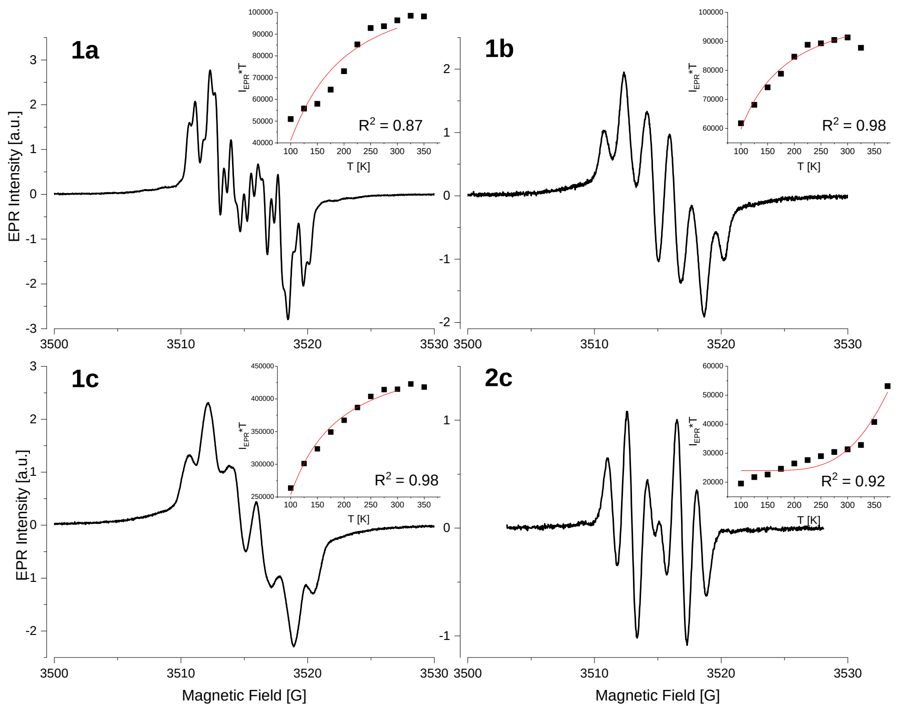

Figure S10c. EPR spectra and VT-EPR data (double integral of intensities*temperature) of **1a-c** and **2c** in toluene and Bleaney-Bowers fit**. 1a-c** degraded after heating over 350 K, while **2c** remained stable.

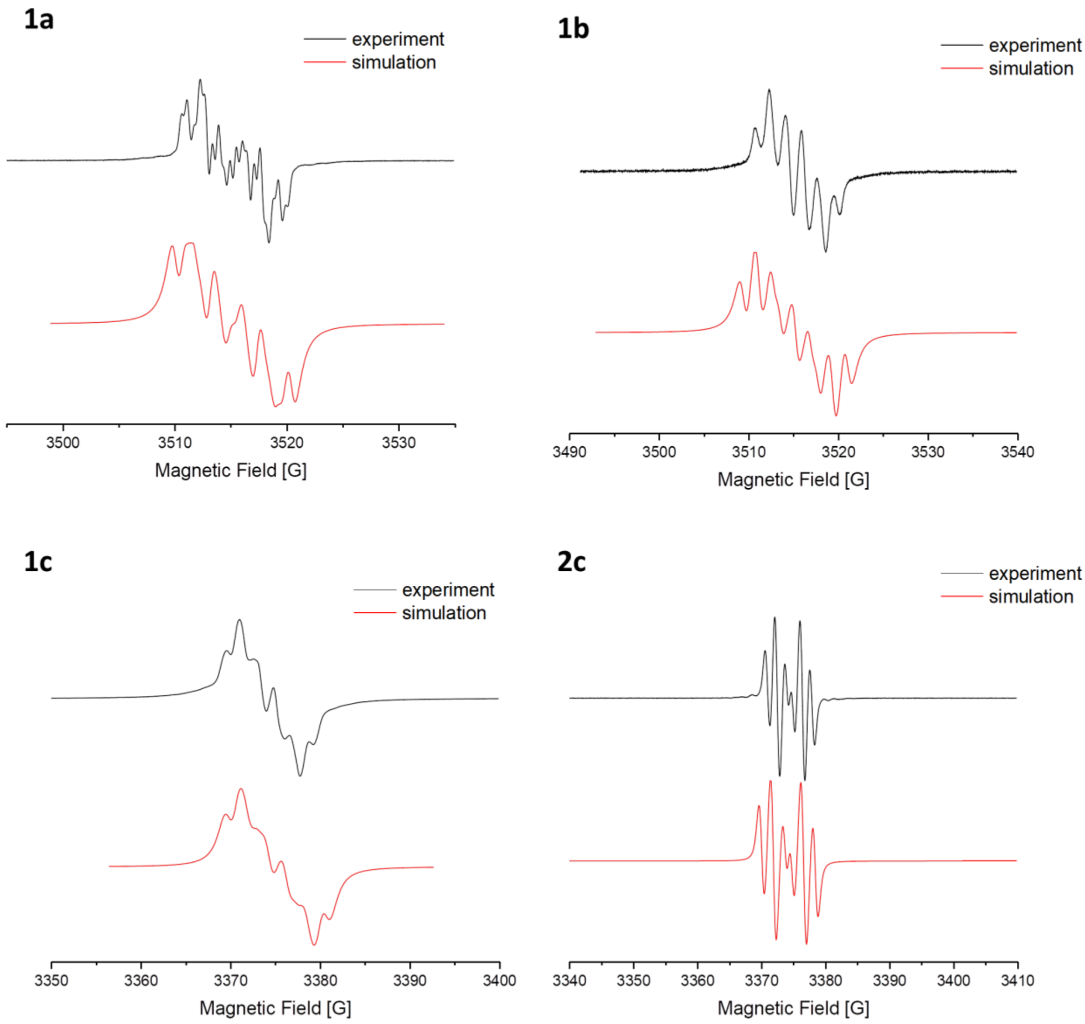

Figure S10d. Comparision between experimental (black) and simulated (red) spectra of **1a-c** and **2c**.

The data of the EPR spectra given in Figure S10d were fitted with the EasySpin simulation package[2] for an isoptropic spectrum (function: *garlic*) assuming no coupling between the electrons (S=1/2) and coupling to the closest ^{1}H and ^{14}N atoms in case of **1a-c** and only to the three closest ^{1}H-atoms for **2c**.

While for **2c** the fine structure can be simulated by taking into account the phenoxyl- and thiophene-protons, for **1a-c** the nitrogen interacts has to be included.. This confirms the theoretical calculations of the spin density distribution which shows the electronic decoupling of the quinoidal system and the azaacene in **2c**.

Table S3. Simulated hfc-coupling constants of **1a-c** and **2c**.

	$^{1}H_a$	$^{1}H_b$	$^{1}H_c$	^{14}N
**1a**	1.800	0.921	5.529	-0.194
	-16.600	7.802	-0.197	6.985
	-20.794	11.073	8.900	0.993
**1b**	-2.120	2.117	7.210	2.674
	-13.3	6.720	2.704	6.784
	-20.600	11.340	4.914	3.900
**1c**	-4.210	1.213	6.399	-0.190
	-12.150	5.481	1.499	7.642
	-18.627	11.067	7.066	3.449
**2c**	-5.789	2.852	2.913	-
	-15.379	8.437	3.605	-
	-18.606	3.34	9.565	-

11. SQUID Magnetometry

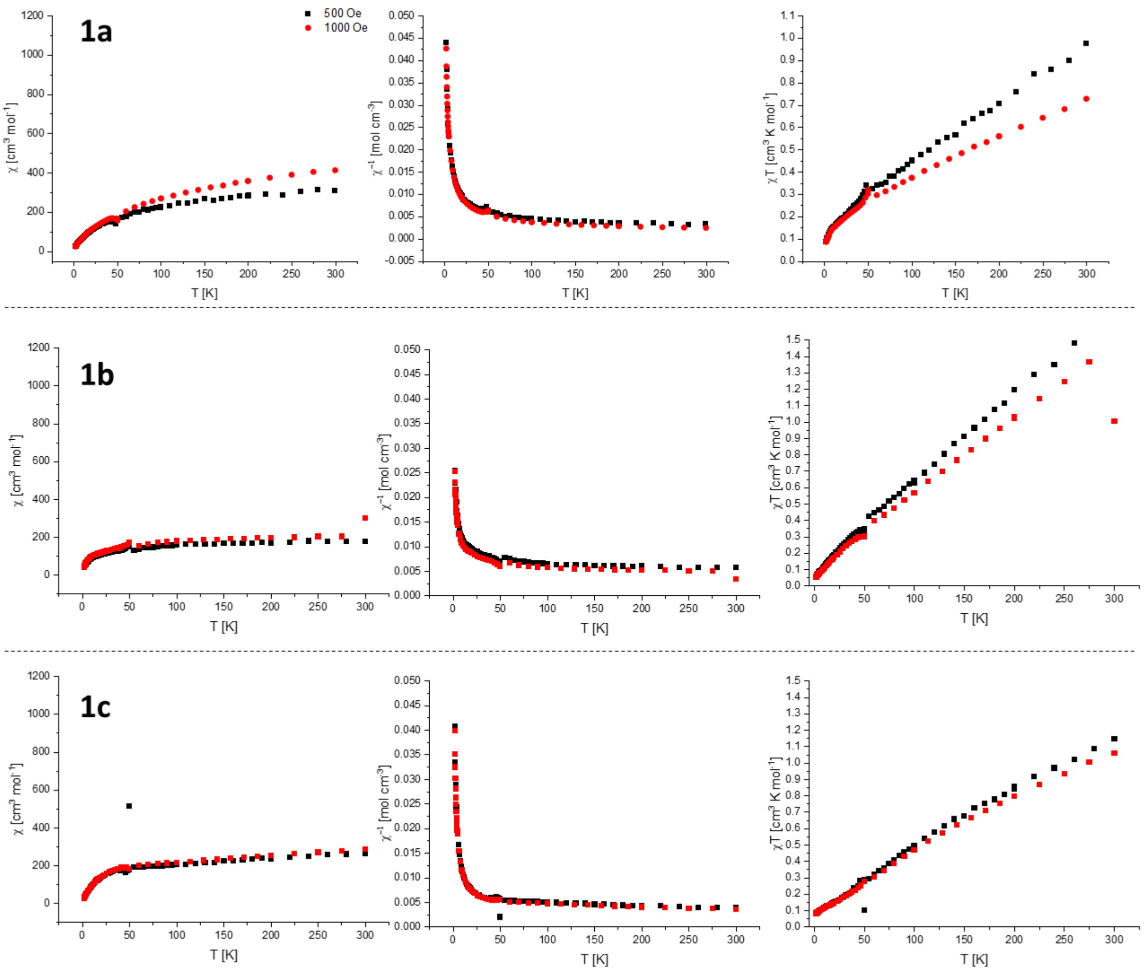

Figure S11a: SQUID measurements from 0-300K in polycarbonate capsules of **1a-c** at 500 and 1000 Oe.

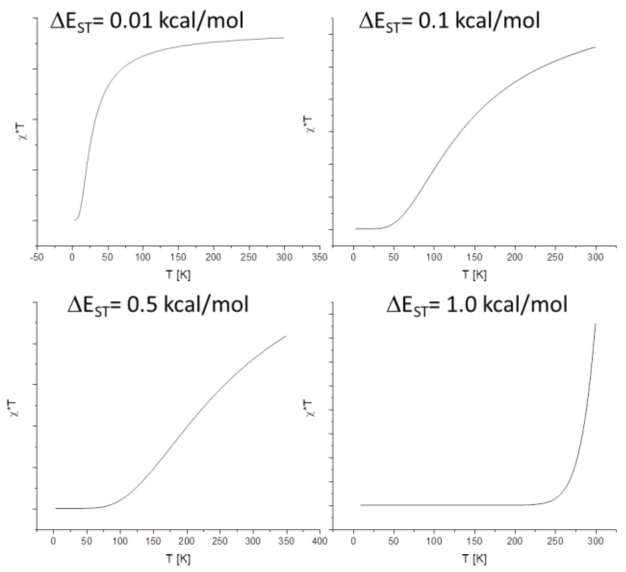

Figure S11b: Simulation of Bleaney-Bowers plots for different ΔE_{ST} gaps.

12. Stability in Toluene under Ambient Conditions

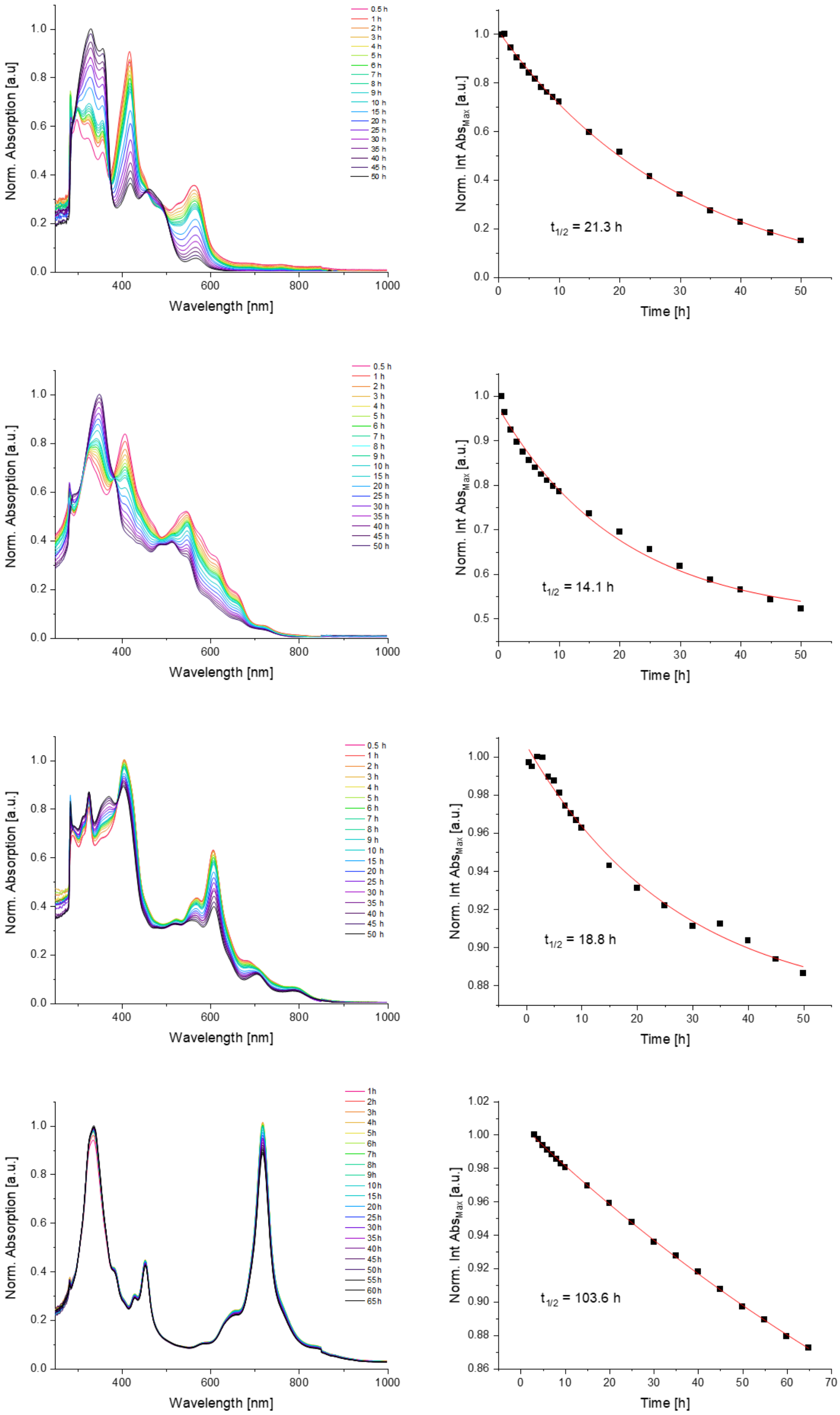

Figure S12 Stability of **1a-c** and **2c** in toluene under ambient conditions.

13. Calculations of Diradical Character

All calculations were carried out using the Gaussian16 program package. The molecules **1a-c** and **2c** (with t-Bu and TIPS groups replaced by Me) were optimized in the closed-shell singlet ground state at the B3LYP/6-311G(d,p) level of theory. Frequency calculations were carried out to confirm the minimum nature of the stationary point.

The diradical character was estimated by performing a natural orbital (NO) population analysis on a state with multiplicity M = 1, where spin symmetry was broken by mixing HOMO and LUMO in the initial guess. From the occupation numbers n of the highest occupied and lowest unoccupied NO (HONO and LUNO) the diradical character y was calculated according to Yamaguchi as

$$y = 1 - \frac{2T}{1+T^2}$$

where $T = \frac{n_{\mathrm{HONO}} - n_{\mathrm{LUNO}}}{2}$.

This procedure was carried out with unrestricted Hartree—Fock (HF) and different density functional theory (DFT) methods employing the standard 6-311G(d,p) basis set. The results are shown in Table S2. It can be observed that in with a larger amount of exact (HF) exchange the calculated value of y generally increases.

Table S4. Calculations of diradical character with different theoretical methods.

	HF	BHLYP	B3LYP	BLYP	**CAM-B3LYP**
**1a**	0.967	0.960	0.955	0.991	**0.956**
**1b**	0.992	0.996	0.949	0.659	**0.993**
**1c**	0.991	0.961	0.866	0.403	**0.978**
**2c**	0.584	0.296	0.036	0.000	**0.260**

14. Evaluation of Structure-Property Relationships Concerning the Diradical Character

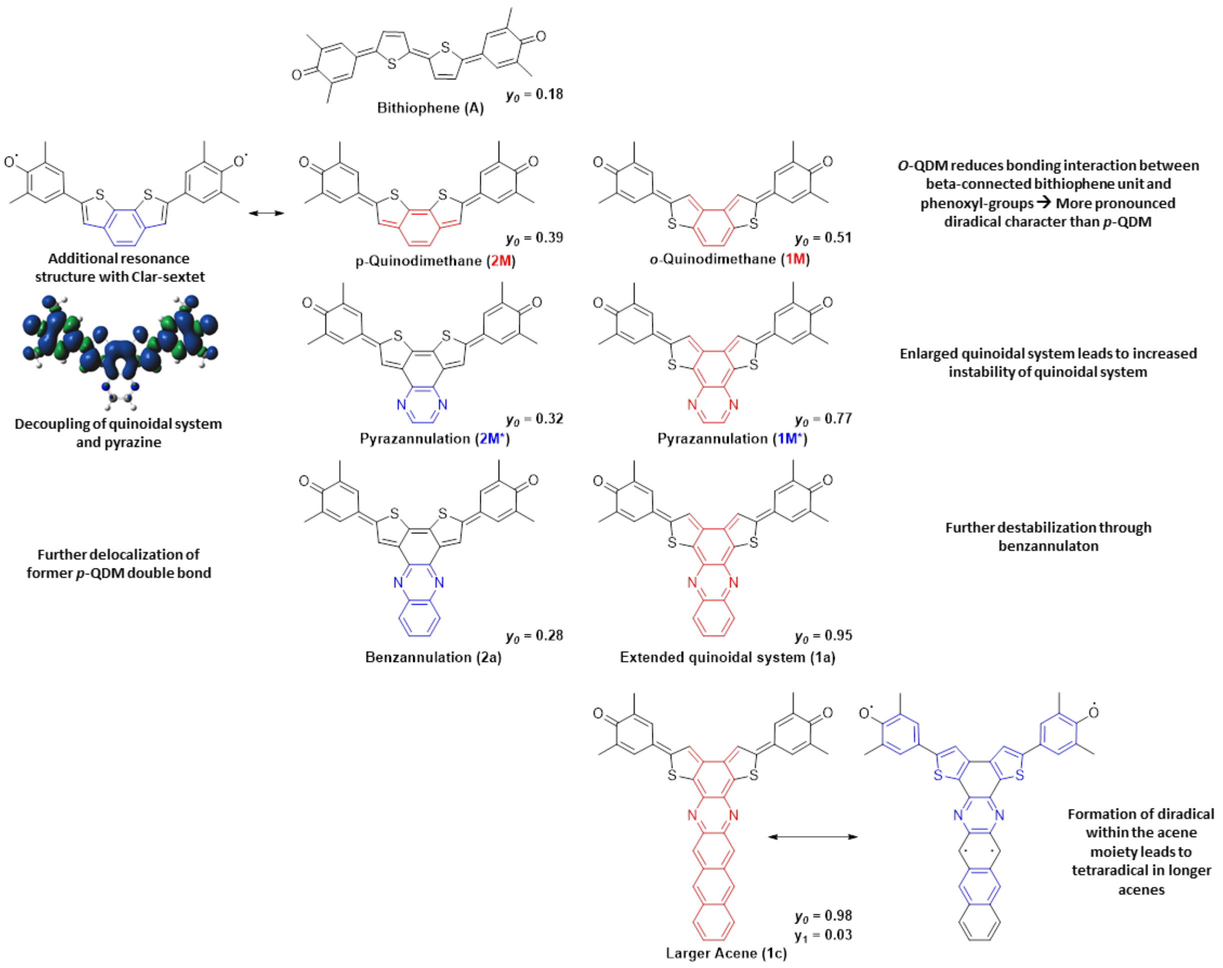

Figure S13. Trend of diradical character y_0 and the singlet-triplet energy gap ΔE_{ST} with increasing acene length (=number of rings). Number of rings equal to zero denotes a non-bridged bisthiophene system. For example, number of rings = 3 for the top regioisomeric series corresponds to non-alkynylated compound **1a**.

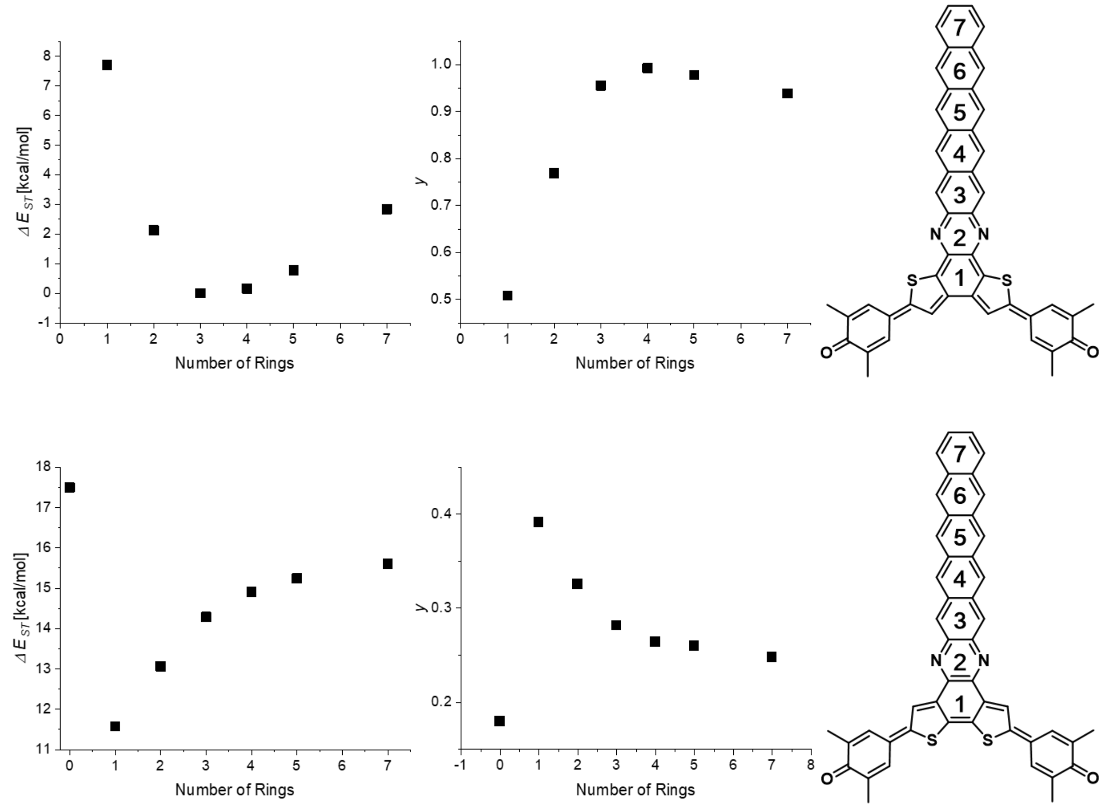

Figure S14. Trend of diradical character y_0 and the singlet-triplet energy gap ΔE_{ST} with increasing acene length.

Table S5. Calculated values of y_0 and ΔE_{ST} with increasing number of annealed rings.

Regioisomer 1

**Number of Rings**	y_0	y_1	ΔE_{ST} [kcal/mol]
**1**	0.51	0.00	7.70
**2**	0.77	0.00	2.13
**3**	0.96	0.00	0.01
**4**	0.99	0.00	0.16
**5**	0.98	0.03	0.77
**7**	0.94	0.12	2.83

Regioisomer 2

**Number of Rings**	y_0	ΔE_{ST} [kcal/mol]
**0**	0.18	17.5
**1**	0.39	11.6
**2**	0.33	13.1
**3**	0.28	14.3
**4**	0.26	14.9
**5**	0.26	15.2
**7**	0.25	15.6

15. HOMA-Calculations

The HOMA (Harmonic Oscillator Model of Aromaticity) is a geometric assessment of aromaticity. For aromatic compounds HOMA approaches 1 while non-aromatic compounds approach 0.

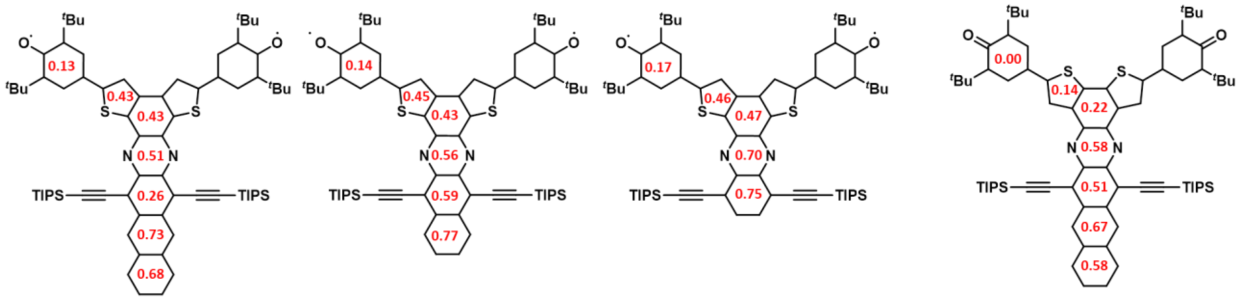

Figure S15. HOMA calculations of the calculated triplet geometries of **1a-c** and singlet CS for **2c**.

HOMA values were calculated according to Dobrowolski *et al.*[1] and Kygowski *et al.*[2,3] with the formula:

$$\boldsymbol{HOMA = 1 - \frac{\alpha}{n}\sum_{i=1}^{n}(R_i - R_{Opt})^2}$$

Where α is a normalization factor of 257.7, n is the number of ring atoms, R_i is the calculated bond length and R_{Opt} is the optimal bond length for aromatic compounds with values of 1.388 for C-C, 1.334 for C-N and 1.677 for C-S bonds.

[1] S. Ostrowski, J. C. Dobrowolski, *RSC Adv.* **2014**, *4*, 44158–44161.

[2] T. M. Krygowski, M. Cyrański, *Tetrahedron* **1996**, *52*, 10255–10264

[3] J. Kruszewski, T. M. Krygowski, *Tetrahedron Lett.* **1972**, *13*, 3839–3842..

16. NICS-XY-Scans

NICS-XY-Scans were calculated 1.7 A above the molecular plane at the B3LYP-6-311 G(++) level of theory.

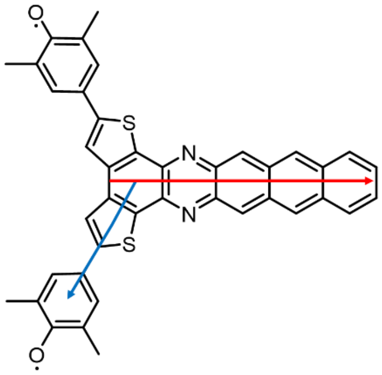

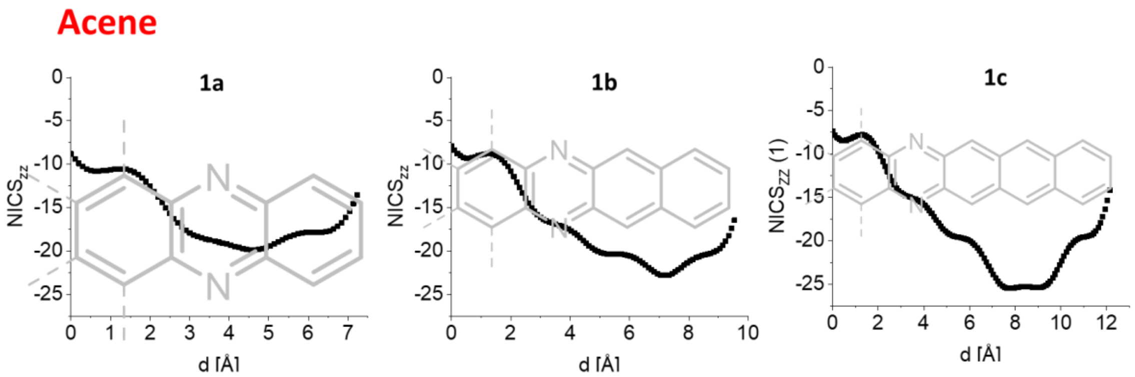

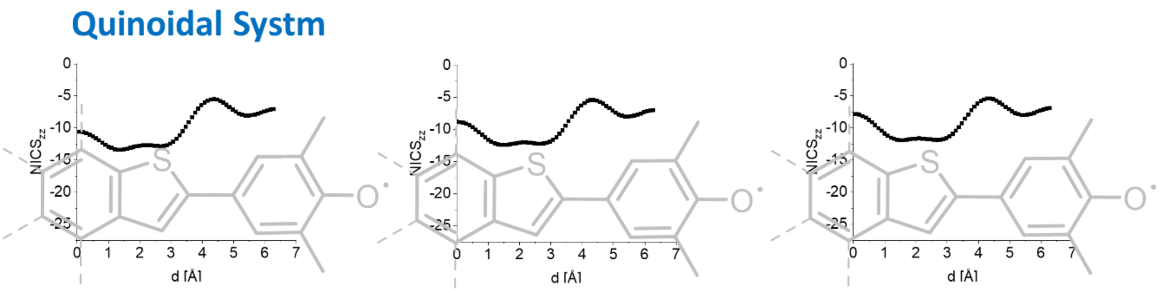

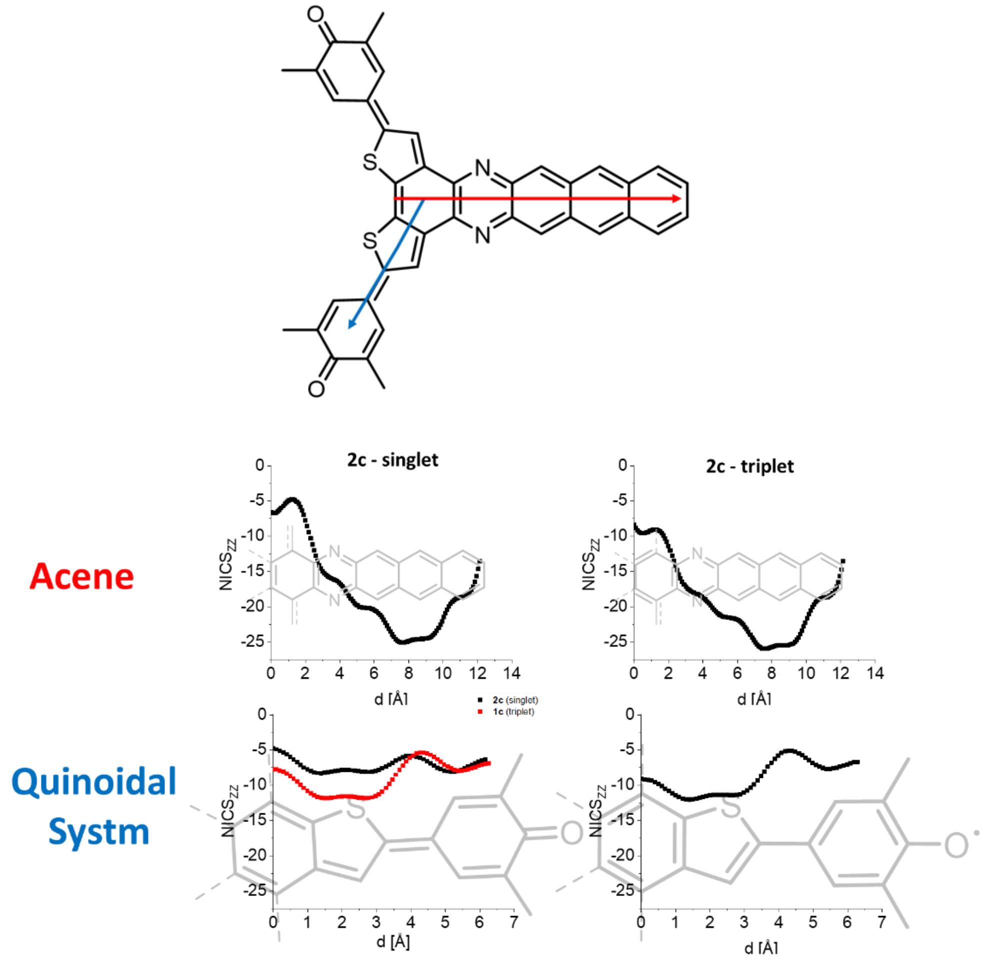

Figure S16. $NICS_{ZZ}$-XY Scans for **1a-c** and **2c**. Scan directions are shown along the acene (red) and along the quinoid system (blue).

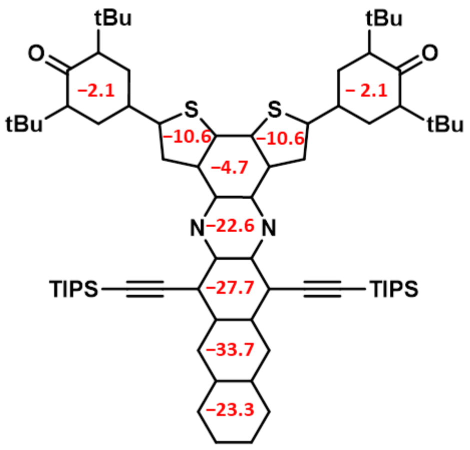

Figure S17. $NICS_{ZZ}$- values for the triplet state of **2c**.

17. General Remarks

All reagents and solvents were obtained from commercial suppliers and were used without further purification. For chromatography, the 40-60°C petroleum ether fraction was used. Preparation of air- and moisture-sensitive materials was carried out in flame-dried flasks under an atmosphere of nitrogen by using Schlenk techniques. For column chromatographical purification a manual column chromatography (SiO_2, grain size 0.04 - 0.063 mm) was used. ^{1}H (^{13}C) NMR-spectra were recorded on 500 MHz (125 MHz) or 600 MHz (150 MHz) spectrometers. Chemical shifts (δ) are reported in parts per million (ppm) relative to traces of [H^1] solvent in the corresponding deuterated solvent. The solvent signals were used as references ($CDCl_3$: δ(^{1}H) = 7.26 and δ (^{13}C) = 77.00 ppm).[1]

HR-MALDI-TOF MS spectra were recorded on a Bruker Autoflex Speed MALDI-TOF MS (Bruker Daltonics, Bremen, Germany) with a *trans*-2-[3-(4-*tert*-Butylphenyl)-2-methyl-2-propenylidene]malononitrile (DCTB) as the matrix. The preparation for all samples was performed in solid state. EI-MS spectra were recorded on a JEOL JMS-700 magnetic sector field instrument.

ATR IR spectra were recorded neat on a Jasco FT/IR-4100 spectrometer and are reportedt in wavenumbers (cm^{-1}).

Pictures were taken with a Canon EOS 7D under daylight or under irradiation with a 254 nm lamp.

Absorption spectra were recorded on a Jasco UV-VIS V-670 spectrophotometer.

Cyclic voltammetry (CV) was performed on a VersaSTAT3–200 potentiostat (Princeton Applied Research) and were carried out using a glassy carbon working electrode, a platinum/titanium wire auxiliary electrode, a silver wire reference electrode, a 0.1 M NBu_4PF_6 solution in degassed dry dichloromethane and ferrocene as an internal standard.

For EPR measurements, an EMXplus X-band CW spectrometer (Bruker BioSpin, Germany) at 100 kHz modulation was used. The EPR spectra were registered at the microwave power of 2 mW. For the precise determination of g-values, a NMR teslameter (ER036TM, Bruker Biospin GmbH) was used. Variable-temperature EPR measurements were performed using an integrated variable temperature controller.

Fitting of VT-EPR-data was performed with the Bleaney-Bowers equation $\chi = \frac{2Ng^2\mu_B^2}{kT\left[3+\exp\left(-\frac{J}{kT}\right)\right]}(1-x) + \frac{Ng^2\mu_B^2}{2kT}\cdot x$

[1] S. Stoll, A. Schweiger, *J. Magn. Reson.* **2006**, *178*, 42–55.

[2] H. E. Gottlieb, V. Kotlyar, A. Nudelman, *J. Org. Chem*. **1997**, 62, 7512-7515.

18. Synthetic Procedures

General procedure for condensation reactions:

The corresponding ortho-diamine (1.00 eq.) and ortho-diketone (1.00 eq) were dissolved in AcOH (10 mL) and stirred at 120 °C for 16 h. The mixture was allowed to cool to room temperature, water and dichloromethane were added and the layers were separated. The aqueous layer was extracted twice with dichloromethane. The combined organic layers were washed with sat. $NaHCO_3$-solution, dried over magnesium sulfate, filtered and the solvent was removed under reduced pressure. The crude products were purified by column chromatography (PE:DCM).

5a: Yield: 16% (55.2 mg, 68.3 µmol). ^{1}H-NMR (500 MHz, $CDCl_3$, 295 K): δ (ppm) = 7.96 (s, 2H), 7.63 (s, 2H), 1.28 (m, 42H).^{13}C-NMR (125 MHz, $CDCl_3$, 295 K): δ (ppm) = 142.29, 138.74, 137.12, 137.06, 136.21, 134.94, 133.64, 126.86, 125.54, 124.14, 120.83, 110.37, 103.61, 100.79, 20.79, 19.83, 12.35, 11.42. IR (neat): $\tilde{\nu}$ = 2939, 2861, 2144, 1533, 1461, 1429, 1416, 1053, 995, 917, 881, 814, 798, 674, 659, 577, 519 cm^{-1}.HRMS (MALDI+): m/z = 808.1014; calcd. for $C_{38}H_{46}Br_2N_2S_2Si_2$ (808.10)

5b: Yield**:** 48% (83.7 mg, 97.5 µmol). ^{1}H-NMR (500 MHz, $CDCl_3$, 295 K): *δ* (ppm) = 8.86 – 8.72 (m, 2H), 7.74 – 7.67 (m, 2H), 7.65 (s, 2H), 1.35 (s, 42H).^{13}C-NMR (125 MHz, $CDCl_3$, 295 K): *δ* (ppm) = 139.51, 139.43, 136.68, 136.03, 128.59, 126.62, 120.62, 120.61, 120.11, 109.87, 107.59, 102.26, 20.48, 19.50, 19.46, 18.53, 12.00. IR (neat): $\tilde{\nu}$ = 2939, 2861, 2349, 2141, 1525, 1459, 1447, 1421, 1403, 1385, 1046, 994, 916, 881, 813, 757, 722, 674, 659, 643, 633, 582, 517, 481, 457, 444, 417. HRMS (MALDI$^+$): *m/z* = 860.1144; calcd. for $C_{42}H_{48}Br_{79}Br_{81}N_2S_2Si_2$ (860.1149).

5c: Yield**:** 59% (104 mg, 114 µmol). ^{1}H-NMR (500 MHz, $CDCl_3$, 295 K): *δ* (ppm) =9.46 (s, 2H), 8.06 (dd, $^3J_{H\text{-}H}$ = 6.5 Hz, $^4J_{H\text{-}H}$ = 3.2 Hz, 2H), 7.63 (s, 2H), 7.53 (dd, $^3J_{H\text{-}H}$ = 6.7 Hz, $^4J_{H\text{-}H}$ = 3.1 Hz, 2H), 1.47 – 1.36 (m, 42H). ^{13}C-NMR (125 MHz, $CDCl_3$, 295 K): *δ* (ppm) = 139.9, 139.4, 136.8, 136.2, 132.9, 132.2, 128.8, 128.6, 126.8, 126.1, 121.0, 120.1, 108.9, 103.3, 19.2, 11.8.IR (neat): $\tilde{\nu}$ = 2938, 2859, 2130, 1527, 1462, 1425, 1370, 1230, 1115, 1031, 873 cm^{-1}.HRMS (MALDI$^+$): *m/z* = 910.1295.calcd. for $C_{46}H_{50}Br_2N_2S_2Si_2$ (910.1300)

8c: Yield**:** 53% (152 mg, 167 µmol). ^{1}H-NMR (500 MHz, $CDCl_3$, 295 K): *δ* (ppm) =9.48 (s,2H), 8.49 (s, 2H), 8.07 (m, 2H), 7.53 (m, 2H), 1.38 (m, 42H). ^{13}C-NMR (125 MHz, $CDCl_3$, 295 K): *δ* (ppm) = 140.1, 139.7, 136.8, 135.5, 133.1, 132.6, 128.9, 128.8, 127.0, 120.8, 113.5, 109.0, 103.7, 19.3, 12.0. IR (neat)**:** $\tilde{\nu}$ = 2938, 2863, 2122, 1460, 1418, 1365, 1210, 1145, 1023, 875 cm^{-1}.HRMS (MALDI$^+$): *m/z* = 912.1903; calcd. for $C_{46}H_{59}N_2Br_2S_2Si_2$ (912.1407).

General procedure for Suzuki couplings:

To a degassed mixture of THF:water (10:1, v/v) was added the dibromo compound (54.9 µmol), 2,4,6-tris[3,5-bis(1,1-dimethylethyl)-4-[(trimethylsilyl)oxy]phenyl]boroxin (49.3 mg, 54.9 µmol), $Pd(PPh_3)_4$ (3.17 mg, 2.74 µmol) and Na_2CO_3 (52.4 mg, 494 µmol) under Ar. The mixture was heated to 70 °C overnight and extracted with ethyl acetate. The combined organic layers were washed with water and brine and dried over $MgSO_4$. The crude product

was purified by column chromatography (silica gel, 20:1 PE:DCM → 5:1 PE:DCM) affording **6a-c** and **9c** as crystalline solids.

6a: Yield: 54% (34.4 mg, 29.7 µmol). ^{1}H NMR (600 MHz, $CDCl_3$) δ = 7.92 (d, J = 1.1 Hz, 2H), 7.88 (d, J = 1.0 Hz, 2H), 7.73 (d, J = 1.0 Hz, 4H), 5.47 (d, J = 1.2 Hz, 2H), 1.58 (s, 37H), 1.36 – 1.29 (m, 42H). ^{13}C NMR (151 MHz, $CDCl_3$) δ = 155.2, 151.8, 142.1, 140.2, 138.7, 137.0, 134.0, 133.4, 126.1, 124.0, 123.9, 117.4, 104.2, 99.9, 34.9, 30.7, 19.4, 11.9. IR (neat) ṽ = 3627, 2941, 2888, 2862, 2156, 1424, 1391, 1239, 1217, 1146, 1054, 1040, 881, 820, 799, 755, 674, 661, 459, 452cm^{-1}. HRMS (MALDI^{+}): m/z = 1110.62; calcd. for $C_{70}H_{90}N_2O_2S_2Si_2$ (1110.492).

6b: Yield: 71% (43.2 mg, 39.0 µmol). ^{1}H NMR (600 MHz, $CDCl_3$) δ = 9.48 (s, 2H), 8.08 (dd, J = 6.5, 3.2 Hz, 2H), 7.85 (s, 2H), 7.75 (s, 4H), 7.52 (dd, J = 6.7, 3.1 Hz, 2H), 5.49 (s, 2H), 1.60 (s, 36H), 1.45 (s, 42H). ^{13}C NMR (151 MHz, $CDCl_3$) δ = 154.8, 151.9, 141.1, 139.8, 138.6, 136.6, 134.4, 133.5, 127.5, 127.4, 125.7, 123.6, 119.7, 117.3, 106.7, 102.9, 34.5, 30.3, 19.1, 11.6. IR (neat) ṽ = 3623, 2955, 2942, 2863, 2369, 2159, 1423, 1391, 1239, 1219, 1149, 881, 819, 799, 675, 661, 439, 432, 415 cm^{-1}. HRMS (MALDI^{+}): m/z = 1110.62; calcd. for $C_{70}H_{90}N_2O_2S_2Si_2$ (1110.492).

6c: Yield: 69% (44.0 mg, 37.9 µmol). ^{1}H NMR (600 MHz, $CDCl_3$) δ = 9.48 (s, 2H), 8.08 (dd, J = 6.5, 3.2 Hz, 2H), 7.85 (s, 2H), 7.75 (s, 4H), 7.52 (dd, J = 6.7, 3.1 Hz, 2H), 5.49 (s, 2H), 1.60 (s, 36H), 1.45 (s, 42H). ^{13}C NMR (151 MHz, $CDCl_3$) δ = 154.9, 152.2, 141.6, 139.7, 138.8, 136.6, 133.5, 132.5, 131.9, 128.6, 126.6, 126.4, 125.6, 123.5, 119.5, 117.5, 107.8, 103.7, 77.2, 77.0, 76.7, 34.5, 30.3, 19.2, 11.7. IR (neat) ṽ = 2962, 2359, 2341,1422, 1275, 1260, 764, 750 cm^{-1}. HRMS (MALDI^{+}): m/z = 1161.565; calcd. for $C_{74}H_{92}N_2O_2S_2Si_2$ (1161.61).

9c: Yield: 85% (54.2 mg, 46.6 µmol). ^{1}H NMR (600 MHz, $CDCl_3$) δ = 9.55 (s, 2H), 8.49 (s, 2H), 8.08 (dd, J = 6.5, 3.2 Hz, 2H), 7.63 (s, 4H), 7.53 (dd, J = 6.6, 3.0 Hz, 2H), 5.43 (s, 2H), 1.57 (s, 36H), 1.41 (d, J = 5.9 Hz, 36H), 0.09 (s, 6H).^{13}C NMR (151 MHz, $CDCl_3$) δ = 154.9, 145.3, 142.2, 139.7, 137.0, 136.4, 136.0, 133.0, 132.6, 129.0, 127.2, 126.9, 126.0, 124.3, 120.5, 112.0, 108.6, 104.5, 77.6, 77.4, 77.2, 34.9, 30.7, 19.5, 12.1. IR (neat) ṽ = 3631, 2954, 2942, 2924, 2863, 2358, 2331, 1424, 1251, 764, 750, 674 cm^{-1}. HRMS (MALDI^{+}): m/z = 1161.562; calcd. for $C_{74}H_{92}N_2O_2S_2Si_2$ (1161.61).

General procedure for the oxidation to yield quinoidal azaacenes:

To a solution of the bisphenols **6a-c** and **9c** (15.0 mg) in 10 mL THF was added 10 mL of a 0.1 M aqueous KOH solution and $K_3[Fe(CN)_6]$ (2.00 eq.). The mixture turned dark immediately and it was stirred for 5 min. The mixture was extracted with ethyl acetate, washed with water and dried over $MgSO_4$. The solvent was removed and the product dried *in vacuo.*

1a: Yield: 91% (13.6 mg, 12.9 µmol) . IR (neat) $\tilde{\nu}$ =2953, 2938, 2922, 2861, 2361, 2342, 1540,1456, 1050, 886, 802, 679, 659 cm^{-1}. HRMS (MALDI$^-$): m/z = 1058.5672; calcd. for $C_{66}H_{86}N_2O_2S_2Si_2$ (1058.5675).

1b: Yield: 86% (12.9 mg, 11.6 µmol). IR (neat) $\tilde{\nu}$ = 2947, 2920, 2864, 1569, 1545, 1533, 1455, 1378, 1357, 1253 cm^{-1}. HRMS (MALDI$^-$)(M+H): m/z = 1109.5898 calcd. for $C_{70}H_{89}N_2O_2S_2Si_2$ (1109.5909).

1c: Yield: 92 % (13 8 mg, 11.9 µmol). IR (neat) $\tilde{\nu}$ = 2950, 2918, 2862, 1541, 1458, 1418, 1360, 1256, 1090, 1025, 879, 801, 727, 673, 662, 457cm^{-1}. HRMS (MALDI$^-$)(M+H): m/z = 1159.6055; calcd. for $C_{74}H_{91}N_2O_2S_2Si_2$ (1159.6066).

2c: Yield: 95% (14.2 mg, 12.3 µmol). ^{1}H NMR (600 MHz, $CDCl_3$) δ = 9.47 (s, 2H), 8.65 (s, 2H), 8.13 – 8.00 (m, 2H), 7.71 – 7.56 (m, 4H), 7.38 (s, 2H), 1.59 – 1.15 (m, 78H). ^{13}C NMR (151 MHz, $CDCl_3$) δ = 141.97, 139.02, 133.23, 132.55, 128.63, 127.27, 127.24, 121.17, 109.92, 103.26, 19.04, 11.56. IR (neat) $\tilde{\nu}$ = 3623, 2939, 2851, 1723,1566, 1453,1358, 1263, 1137, 1088, 1023, 878, 799, 715, 673, 582, 464 cm^{-1}. HRMS (MALDI$^+$)(M+2H): m/z = 1160.604; calcd. for $C_{74}H_{90}N_2O_2S_2Si_2$ (1158.60).

^{1}H NMR ($CDCl_3$, 500 MHz, 293 K) and ^{13}C NMR spectra ($CDCl_3$, 151 MHz, 293 K)

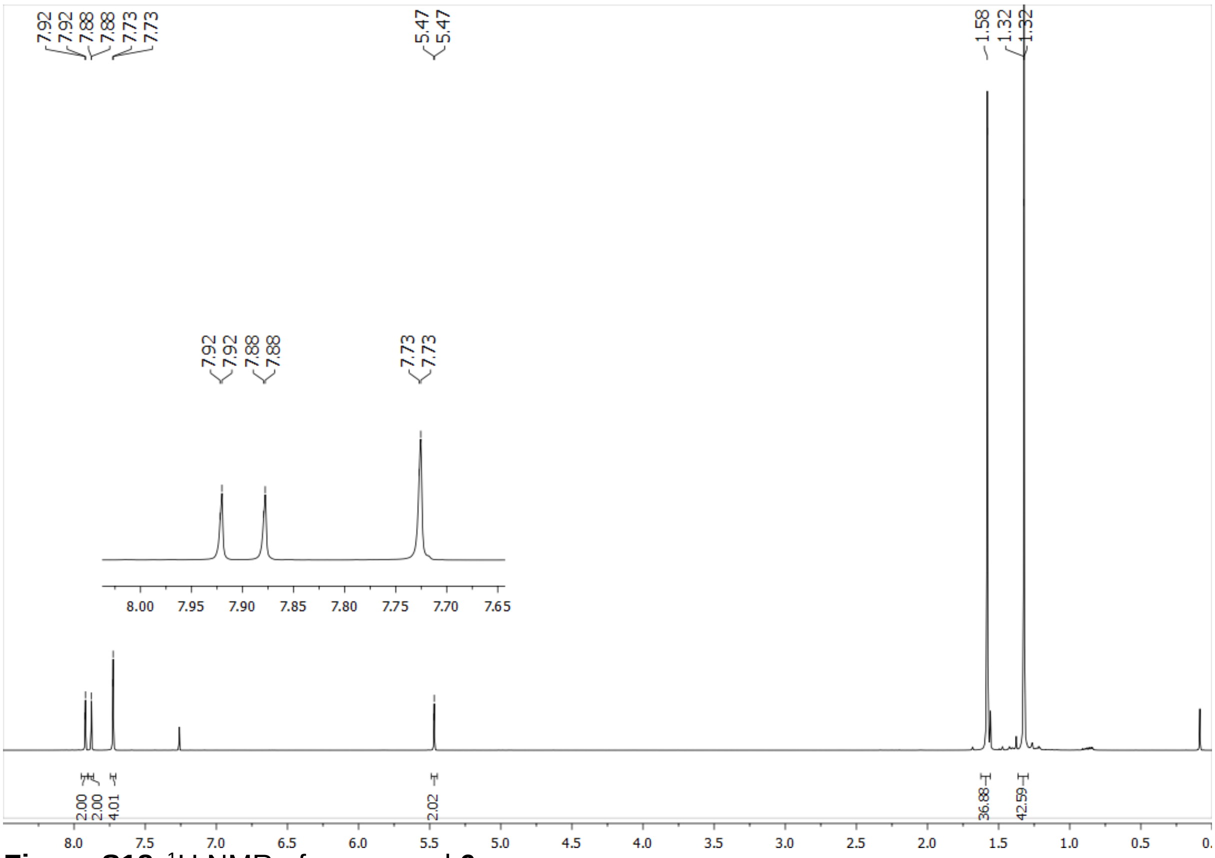

Figure S18: ^{1}H NMR of compound **6a.**

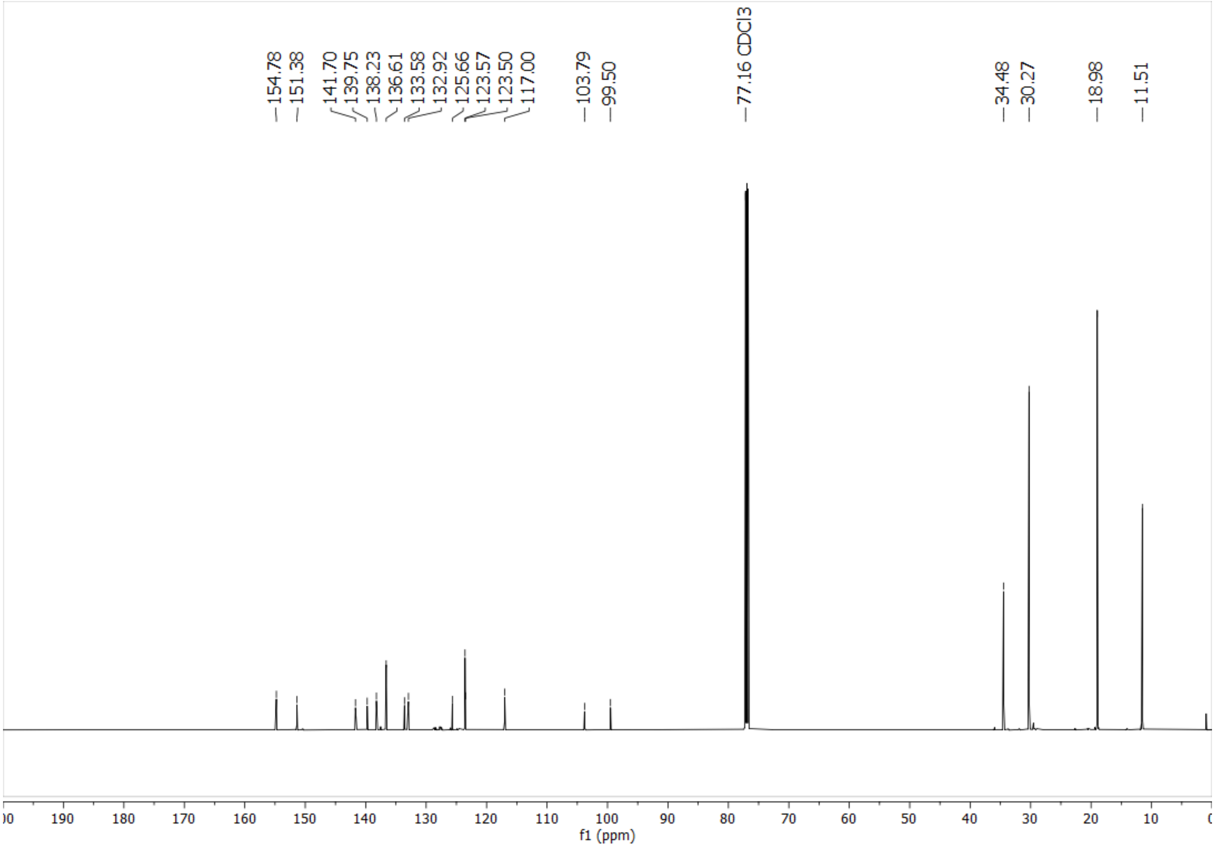

Figure S19: ^{13}C NMR of compound **6a.**

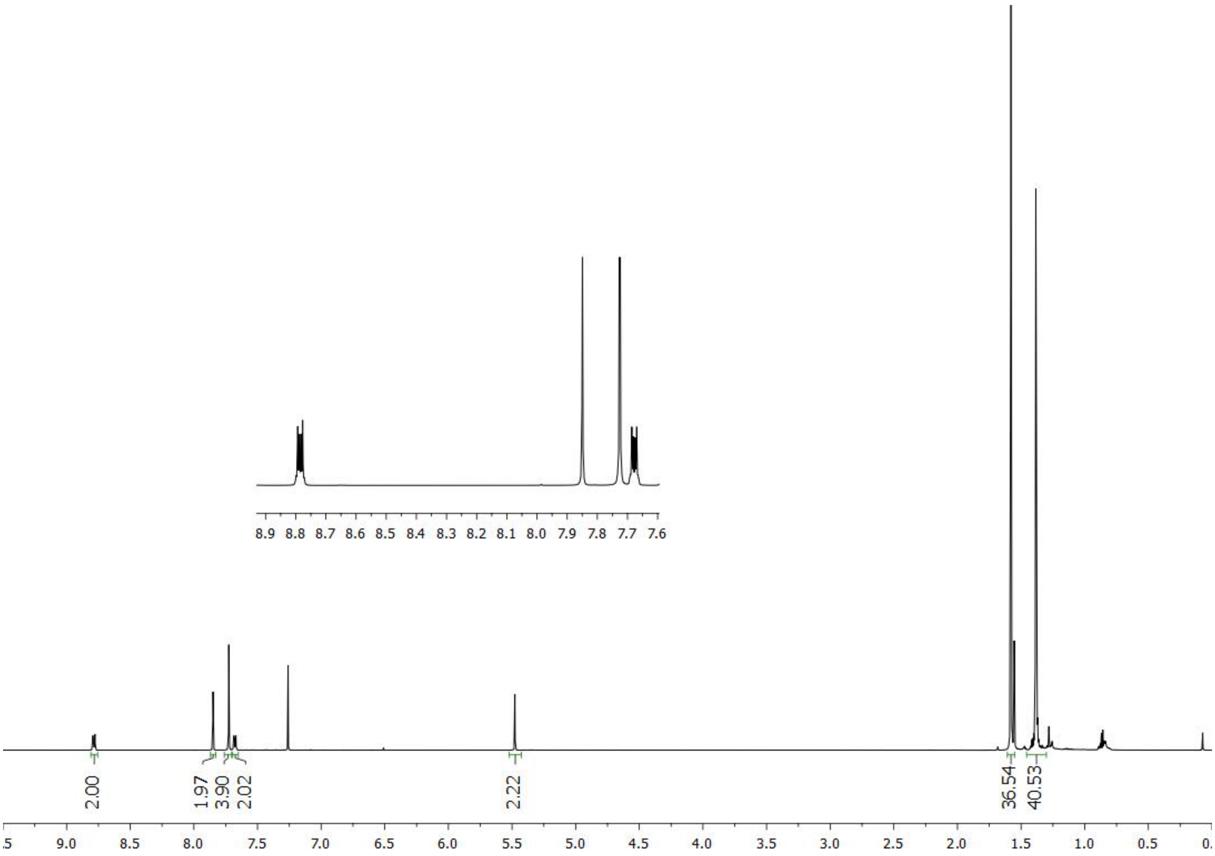

Figure S20: ^{1}H NMR of compound **6b.**

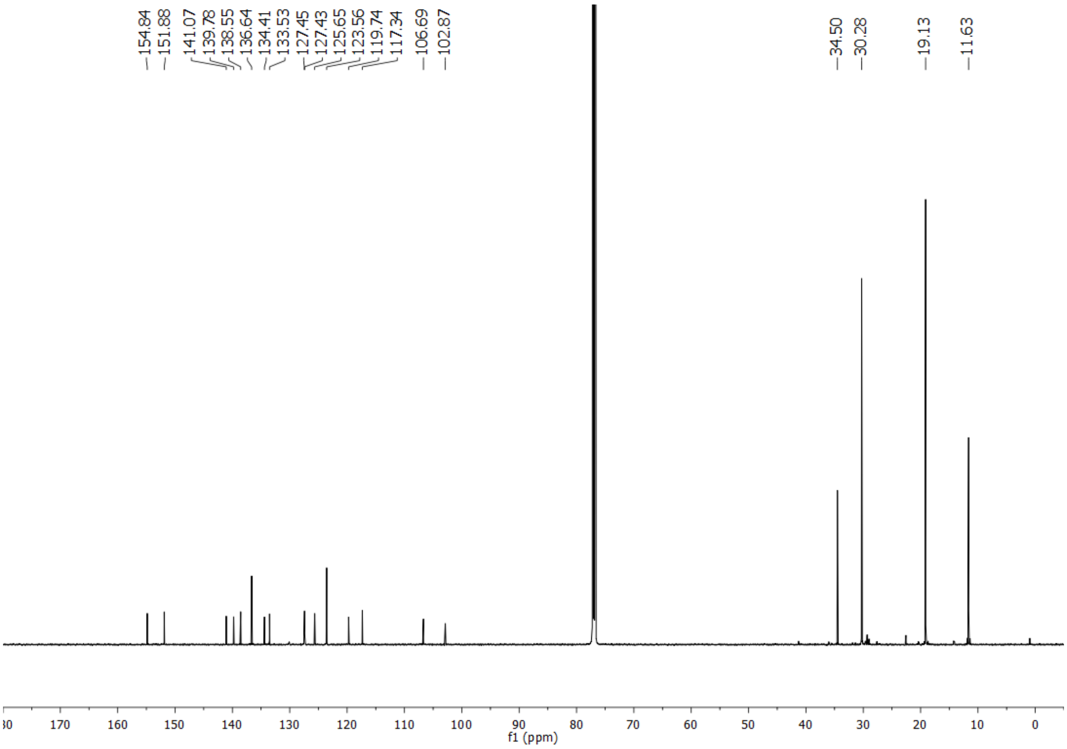

Figure S21: ^{13}C NMR of compound **6b.**

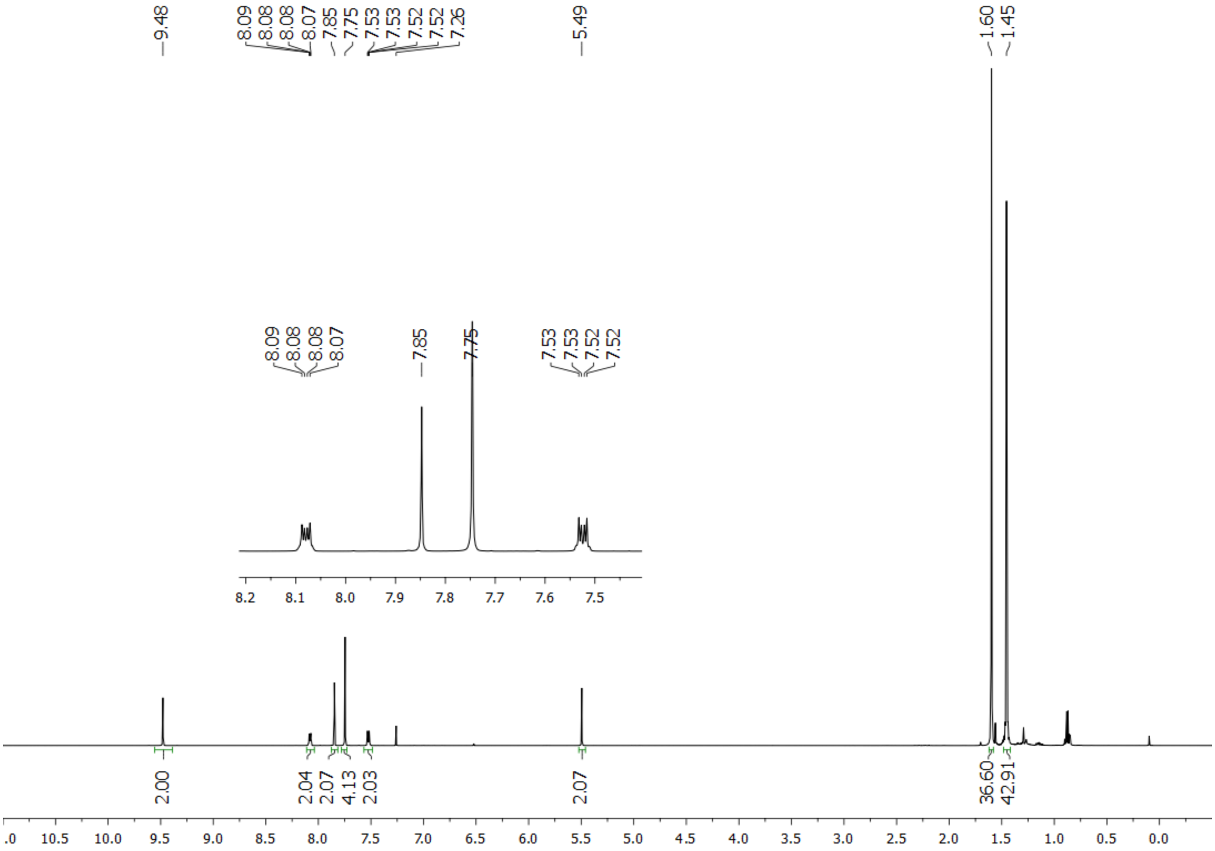

Figure S22: ^{1}H NMR of compound **6c.**

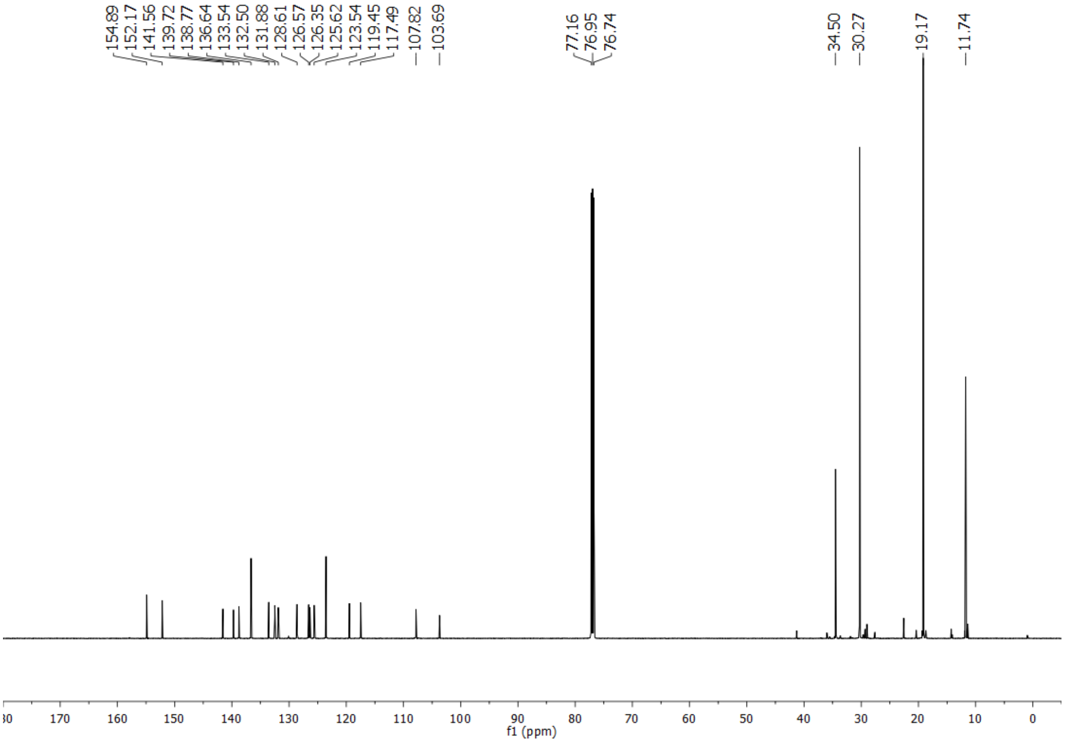

Figure S23: ^{13}C NMR of compound **6c**

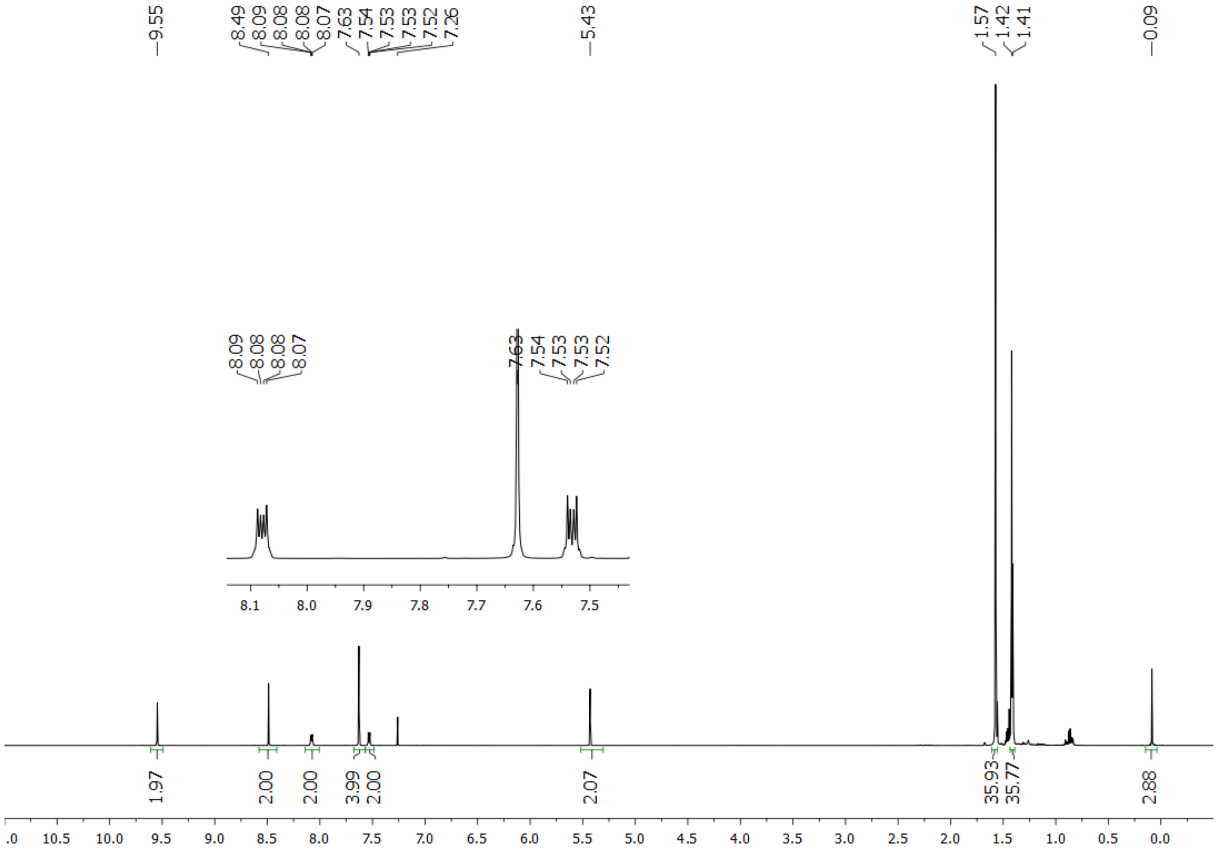

Figure S24: ^{1}H NMR of compound **9c.**

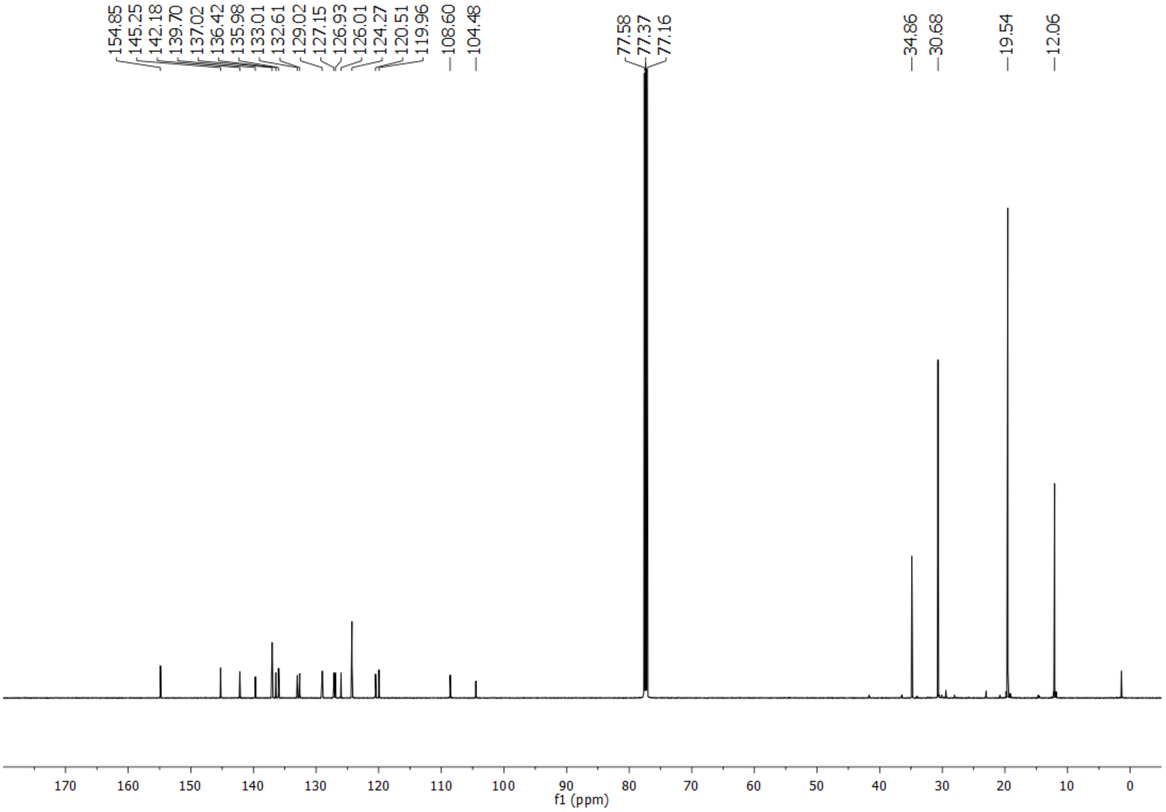

Figure S25: ^{13}C NMR of compound **9c.**

Mass spectra

Mass spectrum (MALDI$^+$) of compound **9c**

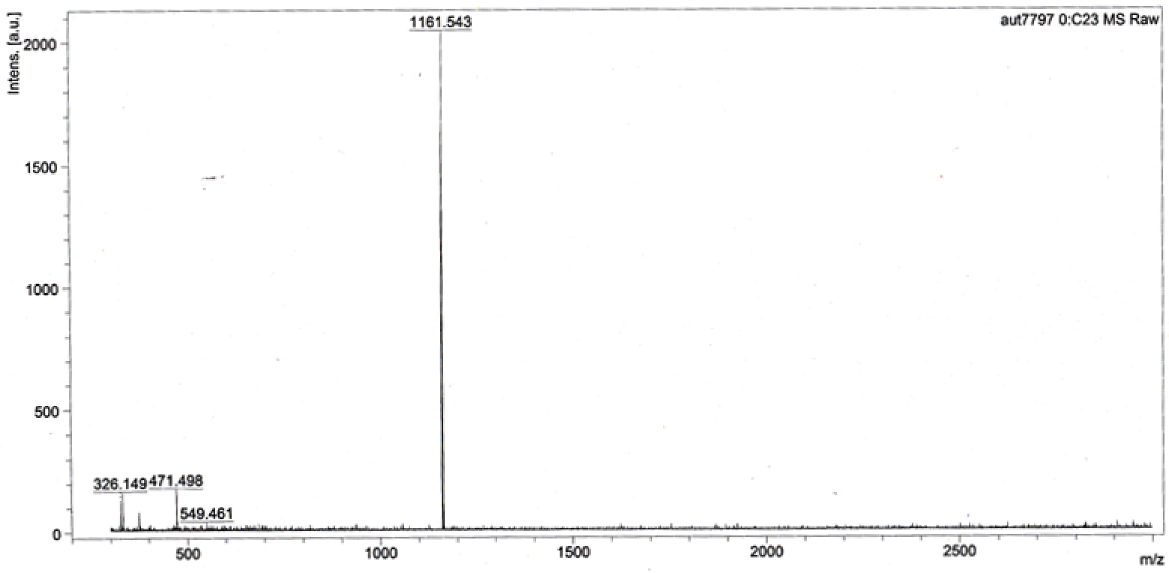

Mass spectrum (MALDI$^+$) of compound **6c**

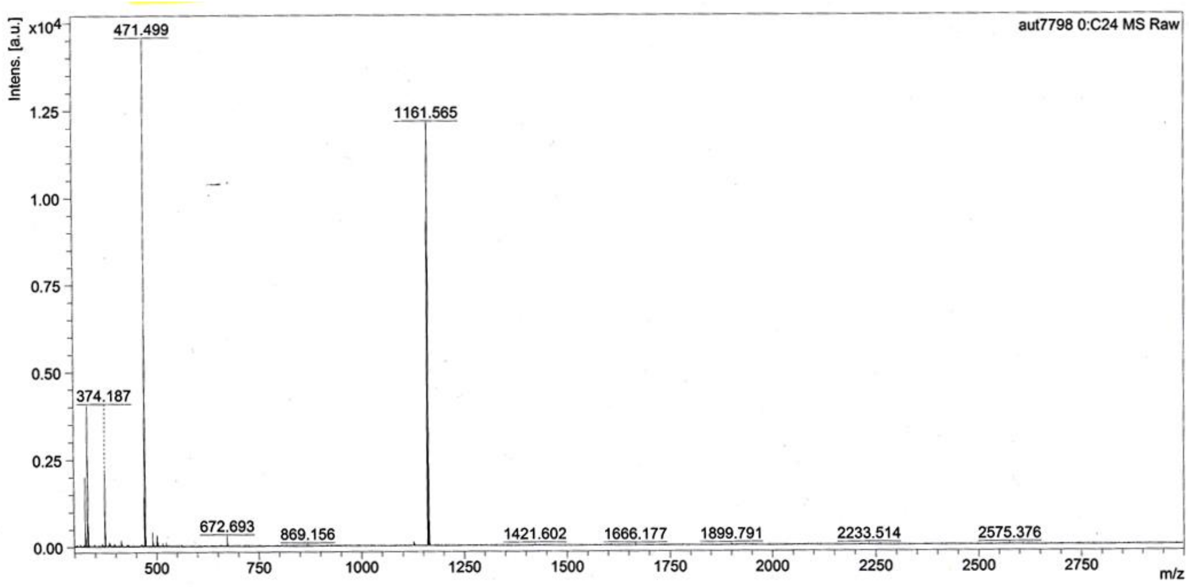

Mass spectrum (MALDI⁻) of compound **2c**

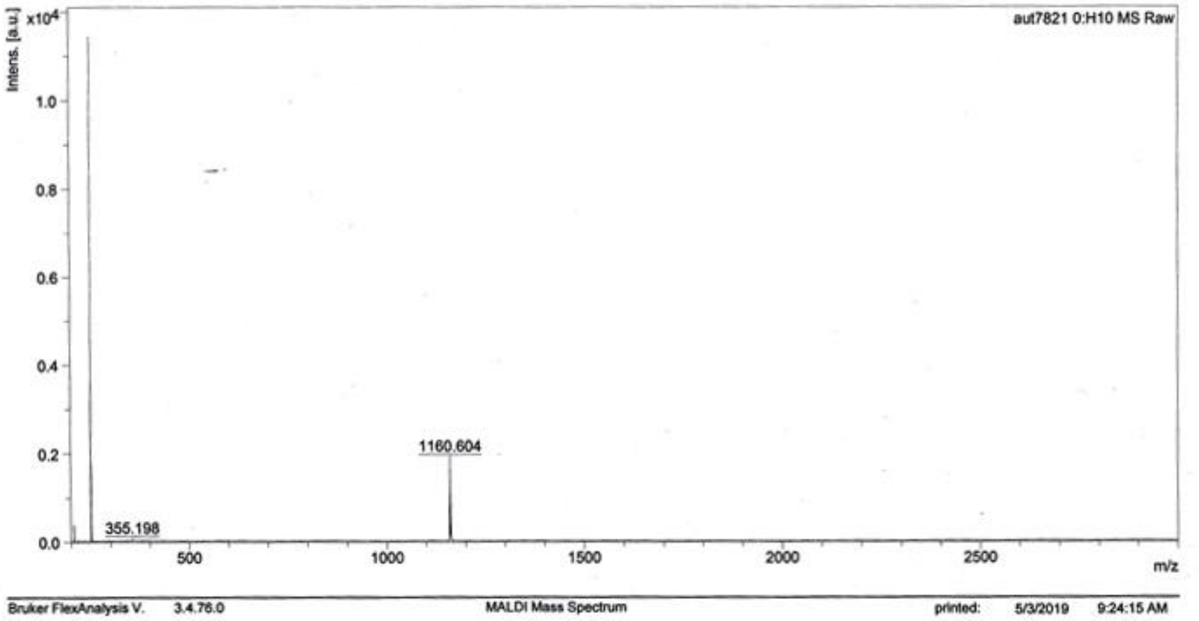

Mass spectrum (MALDI⁻) of compound **1c**

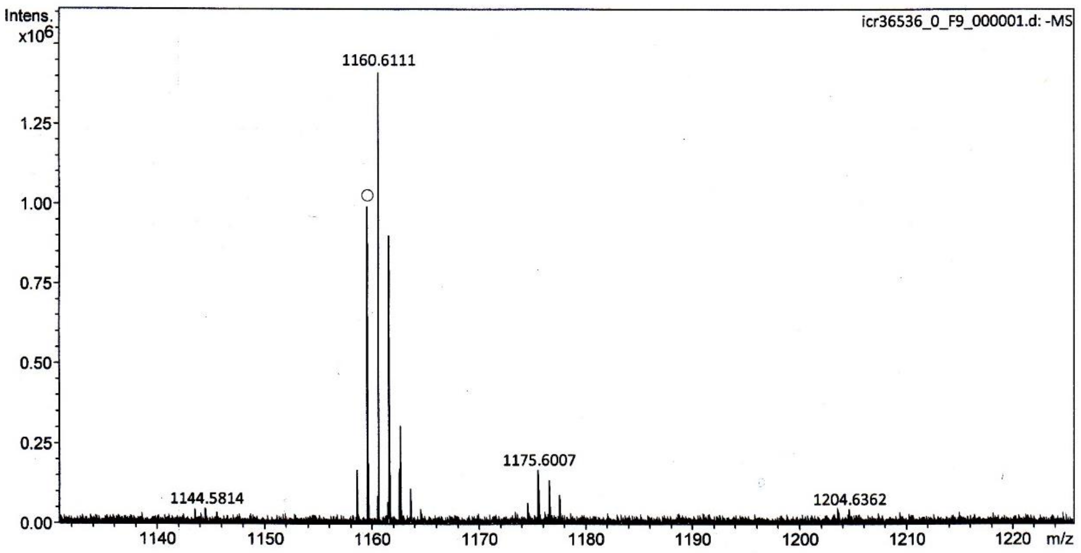

Meas. m/z	Ion Formula	m/z	err [ppm]	mSigma	rdb	e⁻ Conf	N-Rule
1159.6055	C74H91N2O2S2Si2	1159.6066	1.0	138.1	32.5	even	ok

Mass spectrum (MALDI$^-$) of compound **6b**

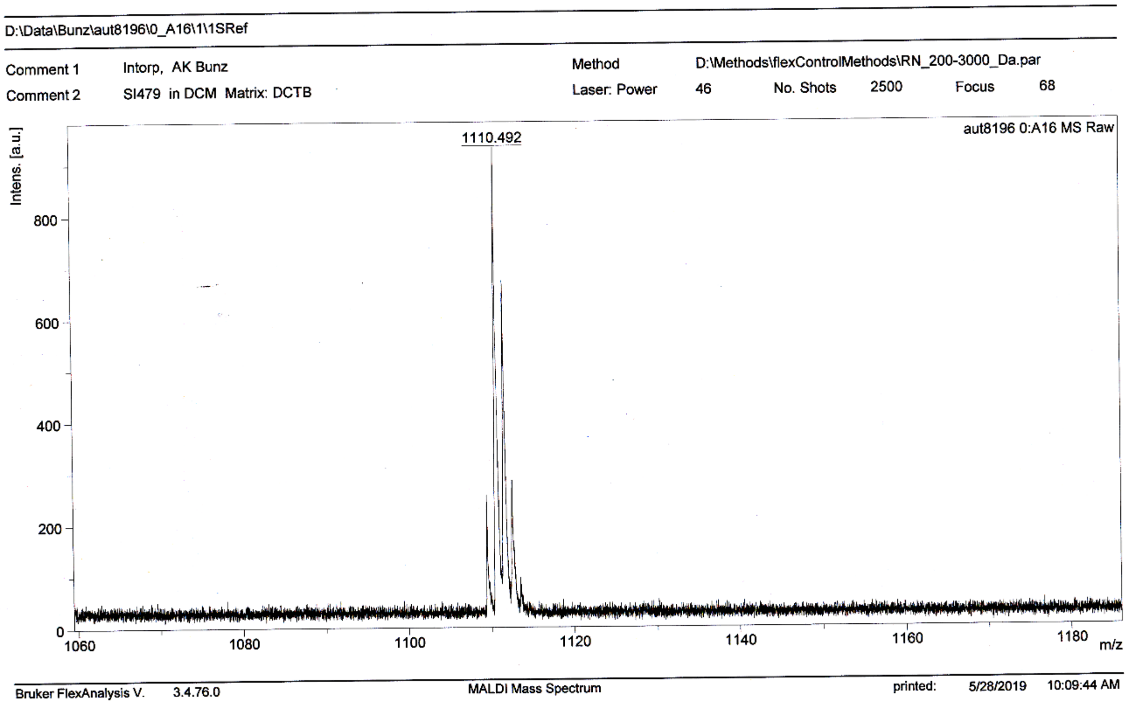

Mass spectrum (MALDI$^-$) of compound **1b**

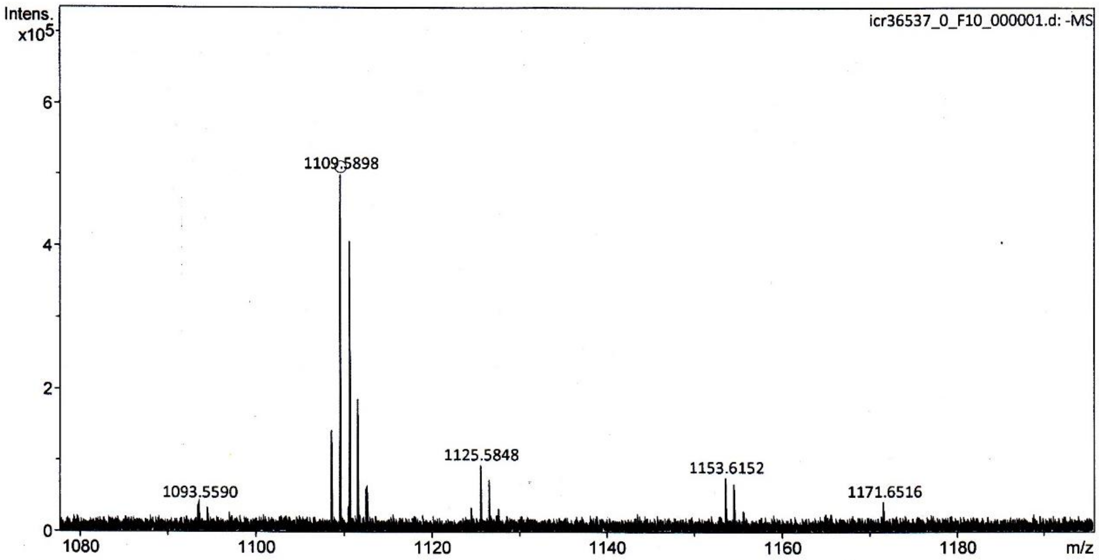

Meas. m/z	Ion Formula	m/z	err [ppm]	mSigma	rdb	e⁻ Conf	N-Rule
1109.5898	C70H89N2O2S2Si2	1109.5909	1.0	101.7	29.5	even	ok

Mass spectrum (MALDI⁻) of compound **6a**

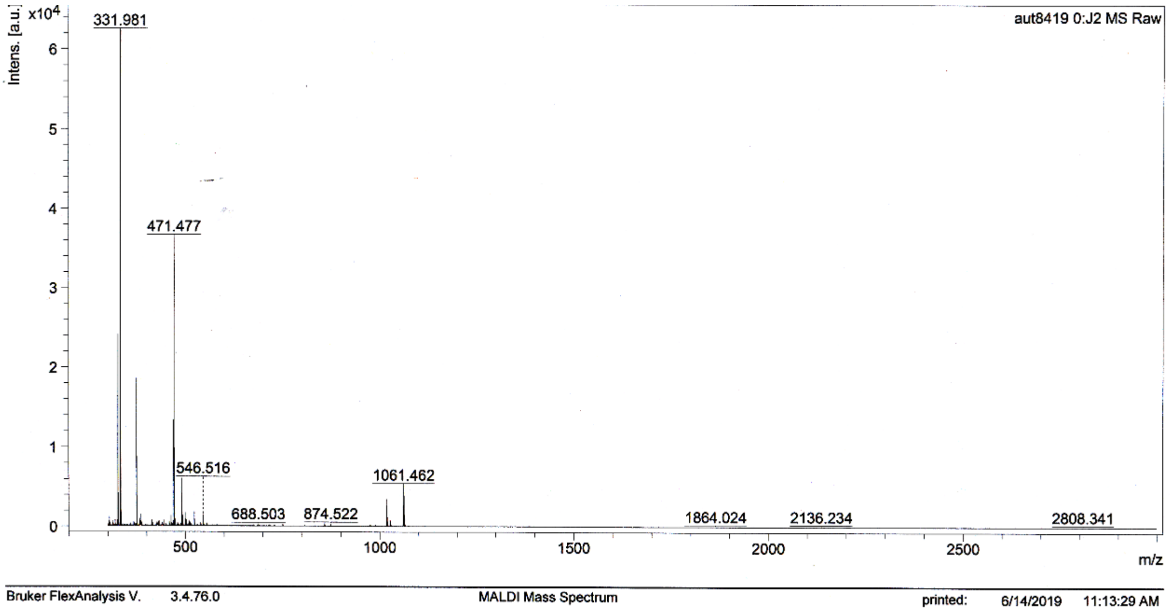

Mass spectrum (MALDI⁻) of compound **1a**

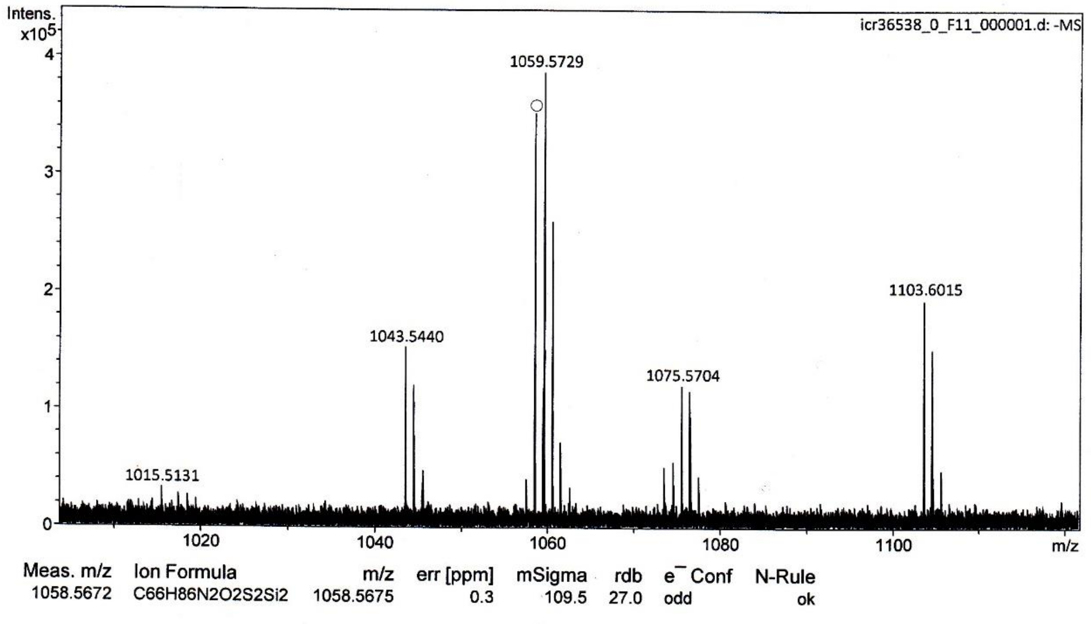

21. Crystallographic data

Table S6: Crystal data and structure refinement for **1c**

Empirical formula	$C_{83}H_{101}N_3O_2S_2Si_2$	
Formula weight	1292.96	
Temperature	200(2) K	
Wavelength	1.54178 Å	
Crystal system	monoclinic	
Space group	$P2_1/c$	
Z	4	
Unit cell dimensions	a = 18.8940(13) Å	α = 90 deg.
	b = 11.5336(6) Å	β =102.951(5) deg.
	c = 35.252(2) Å	γ = 90 deg.
Volume	7486.6(8) $Å^3$	
Density (calculated)	1.15 g/cm^3	
Absorption coefficient	1.31 mm^{-1}	
Crystal shape	plank	
Crystal size	0.141 x 0.042 x 0.030 mm^3	
Crystal colour	purple	
Theta range for data collection	3.9 to 43.5 deg.	
Index ranges	$-16 \le h \le 16$, $-8 \le k \le 10$, $-31 \le l \le 31$	
Reflections collected	31685	
Independent reflections	5548 (R(int) = 0.1611)	
Observed reflections	2998 ($I > 2\sigma(I)$)	
Absorption correction	Semi-empirical from equivalents	
Max. and min. transmission	1.51 and 0.72	
Refinement method	Full-matrix least-squares on F^2	
Data/restraints/parameters	5548 / 2593 / 1016	
Goodness-of-fit on F^2	1.06	
Final R indices (I>2sigma(I))	R1 = 0.072, wR2 = 0.143	
Largest diff. peak and hole	0.29 and -0.22 $eÅ^{-3}$	

Table S7: Crystal data and structure refinement for **2c.**

Empirical formula	$C_{74}H_{90}N_2O_2S_2Si_2$	
Formula weight	1159.77	
Temperature	200(2) K	
Wavelength	1.54178 Å	
Crystal system	monoclinic	
Space group	$P2_1/c$	
Z	4	
Unit cell dimensions	a = 14.6791(14) Å	α = 90 deg.
	b = 36.738(3) Å	β =100.057(8) deg.
	c = 12.5846(12) Å	γ = 90 deg.
Volume	6682.4(11) $Å^3$	
Density (calculated)	1.15 g/cm^3	
Absorption coefficient	1.41 mm^{-1}	
Crystal shape	plank	
Crystal size	0.170 x 0.040 x 0.018 mm^3	
Crystal colour	green	
Theta range for data collection	3.3 to 47.8 deg.	
Index ranges	$-10 \le h \le 14$, $-35 \le k \le 35$, $-11 \le l \le 12$	
Reflections collected	23405	
Independent reflections	6146 (R(int) = 0.1581)	
Observed reflections	2965 ($I > 2\sigma(I)$)	

Absorption correction	Semi-empirical from equivalents
Max. and min. transmission	1.39 and 0.80
Refinement method	Full-matrix least-squares on F^2
Data/restraints/parameters	6146 / 674 / 763
Goodness-of-fit on F^2	1.03
Final R indices (I>2sigma(I))	R1 = 0.074, wR2 = 0.100
Largest diff. peak and hole	0.28 and -0.24 eÅ^{-3}
